# A Melittin-Derived Lead Compound Ameliorates Severe Acute Pancreatitis by Restoring Oxidative Homeostasis and Macrophage Metabolism

**DOI:** 10.1007/s10753-025-02444-9

**Published:** 2026-01-22

**Authors:** Xiaolong Chen, Ya Chen, Yunyun Mao, Xinxin Chen, Yilin Zhou, Jianfeng Tu

**Affiliations:** 1 Emergency and Critical Care Center, Department of Emergency Medicine, Zhejiang Provincial People’s Hospital, Affiliated People’s Hospital, Hangzhou Medical College, Hangzhou, 310014 PR China; 2 College of Pharmacy, Zhejiang University of Technology, Hangzhou, 310014 PR China; 3 Second Clinical Medical College, Hangzhou Normal University, Hangzhou, 310014 PR China

**Keywords:** Severe acute pancreatitis, Macrophage oxidative stress, Melittin, Anti-inflammatory activity, Metabolic reprogramming

## Abstract

Severe acute pancreatitis (SAP) is a life-threatening inflammatory condition driven by macrophage-mediated oxidative stress and metabolic dysregulation. While bioactive peptides such as melittin show anti-inflammatory potential, their clinical application is limited by cytotoxicity and unclear mechanisms. In this study, we developed HMLT, a melittin-derived peptide with histidine substitutions designed to reduce cytotoxicity. Compared with native melittin, HMLT exhibited significantly lower cytotoxicity in RAW264.7 macrophages while maintaining potent anti-inflammatory activity, as demonstrated by reduced TNF-α release and downregulated expression of TNF-α, IL-6 and IL-1β. Flow cytometry analysis revealed that HMLT reduced ROS accumulation and protected mitochondrial membrane potential in LPS-stimulated macrophages. Additionally, HMLT decreased nitric oxide release and suppressed inducible nitric oxide synthase expression. Metabolomic analysis showed that HMLT restored metabolic balance by increasing endogenous antioxidants including O-acetylcarnitine and ornithine, while downregulating glycolytic intermediates such as phosphoenolpyruvic acid, 2-phospho-D-glyceric acid and 3-phosphoglyceric acid. In a caerulein and LPS-induced murine SAP model, HMLT administration significantly alleviated pancreatic injury, as evidenced by reduced serum amylase and lipase levels, diminished edema. Further mechanistic studies revealed that HMLT inhibited TNF-α secretion and suppressed PKM2-mediated glycolysis in M2-like macrophages. Collectively, these findings demonstrate that HMLT overcomes the toxicity limitations of native melittin and ameliorates SAP through coordinated restoration of oxidative homeostasis and metabolic reprogramming in macrophages, highlighting its promise as a lead compound for SAP treatment.

**Supplementary Information:**

The online version contains supplementary material available at 10.1007/s10753-025-02444-9.

## Introduction

Severe acute pancreatitis (SAP) is a critical inflammatory disorder with substantial global morbidity, exhibiting an annual incidence of 30–40 cases per 100,000 individuals [1, 2]. Approximately 20% of patients with acute pancreatitis (AP) progress to SAP, which is characterized by persistent organ failure and carries a mortality rate as high as 20–40%, making it one of the most severe gastrointestinal emergencies [3, 4]. The severity of SAP stems from its pathological progression: starting as uncontrolled local inflammation, it develops into systemic inflammatory response syndrome (SIRS), which ultimately drives multi-organ dysfunction [3–5]. Current clinical management remains predominantly supportive, focusing on fluid resuscitation, nutritional support, and complication management [4, 6]. However, these approaches essentially constitute passive, late-stage remediation for already impaired organs, failing to effectively interrupt the self-amplifying “inflammatory storm” at the core of SAP pathogenesis. Therefore, developing innovative strategies capable of precisely targeting specific inflammatory mechanisms represents an urgent, unmet clinical need.

Central to the dysregulated immune response in SAP are monocytes and macrophages [7, 8]. Modern pathological studies confirm that the core pathogenesis stems from excessive innate immune activation triggered by tissue injury. Etiological factors (e.g., gallstones, hyperlipidemia) induce acinar cell injury and necrosis, leading to the release of damage-associated molecular patterns (DAMPs) like high mobility group box 1 (HMGB1) and mitochondrial DNA, which potently activate innate immune cells including macrophages via pattern recognition receptors such as Toll-like receptors, promoting their massive infiltration into pancreatic tissue [9, 10]. Within this milieu, infiltrated monocytes differentiate into macrophages, which polarize into two main functional states: the pro-inflammatory M1 phenotype and the anti-inflammatory M2 phenotype [11–13]. A persistent predominance of the M1 phenotype is clinically correlated with exacerbated tissue injury, organ failure, and poor prognosis. These activated M1-like macrophages orchestrate injury through two interconnected and self-amplifying pathways: (1) the secretion of a potent cascade of pro-inflammatory cytokines, including tumor necrosis factor-alpha (TNF-α), interleukin-1beta (IL-1β), and interleukin-6 (IL-6), which drives the characteristic systemic “inflammatory storm” and contributes to remote organ dysfunction [14]; and (2) the excessive generation of reactive oxygen and nitrogen species (ROS/RNS) [15, 16].

Critically, the overproduction of ROS/RNS induces significant oxidative stress, which promotes further apoptotic or necrotic cell death, releasing additional DAMPs. These newly released DAMPs, in turn, activate nuclear factor kappa-B (NF-κB) signaling and the NOD-like receptor thermal protein domain associated protein 3 (NLRP3) inflammasome, leading to enhanced secretion of TNF-α and IL-1β [17, 18]. These cytokines then stimulate further ROS production, thereby establishing a vicious and self-amplifying cycle of “inflammation-oxidative stress-cell death” that is central to the propagation and perpetuation of both local pancreatic and systemic injury in SAP [19, 20].

The oxidative stress can further induce metabolic reprogramming of macrophages, thereby affecting their function and polarization. M1 macrophages primarily rely on glycolysis to meet their rapid energy demands and support inflammatory responses while M2 macrophages mainly depend on oxidative phosphorylation (OXPHOS) for energy. Under oxidative stress conditions, transcription factors like hypoxia inducible factor-1α (HIF-1α) are activated, promoting the expression of glycolysis-related genes, thereby driving macrophages towards M1 polarization [21]. ROS can also activate cell cycle checkpoint kinase 2 (Chk2), which phosphorylates pyruvate kinase M2 (PKM2), promoting glycolysis and facilitates macrophage M1 polarization [22]. This oxidative stress-mediated metabolic shift reinforces the pro-inflammatory milieu, making the disruption of this cycle a compelling therapeutic strategy.

Modulating macrophage oxidative stress to rebalance the pro-inflammatory activity and anti-inflammatory activity holds great potential for improving inflammation. For instance, Li et al. developed biomimetic nanozymes-PPFeCs that scavenge ROS to mitigate oxidative colonic damage and shift macrophage glucose metabolism via the phosphatidylinositol 3-kinase (PI3K)/protein kinase B (Akt) pathway, thereby suppressing inflammatory bowel disease progression [23]. Similarly, Cheng et al. created Pd@M nanocarriers with a Pd core and macrophage-derived vesicle shell for ulcerative colitis treatment, which scavenge ROS, regulate macrophage polarization by inhibiting glycolysis, and reduce neutrophil infiltration [24]. However, these strategies rely on the construction of complex nanosystems, which may limit their applications due to the intricate synthesis and potential challenges in large-scale production.

Venom-based therapies like bee venom have long been utilized in traditional Chinese medicine, where they align with the “using toxins to combat toxins” principle to dispel wind, unblock collaterals, and alleviate pain through blood activation. Modern research is progressively unraveling the molecular mechanisms underlying their anti-inflammatory effects, providing new interpretations for the scientific basis of these traditional practices. For example, mastoparan M from wasp venom inhibits the mitogen-activated protein kinases (MAPK)/NF-κB pathway and suppresses NLRP3 activation, thereby alleviating gouty arthritis [25]. Similarly, LyTx-Pa2a, derived from spider venom, downregulates inflammatory mediators in macrophages [26], while melittin (MLT) from bee venom exhibits broad therapeutic activity across dermatological, neurological, and autoimmune disorders [27–29]. The simplicity and scalability of peptide-based therapeutics offer distinct practical advantages. However, the clinical application of many bioactive peptides is limited by dose-dependent cytotoxicity, which results in a narrow therapeutic window and can lead to unintended cell death at higher concentrations. Moreover, the mechanisms of action of these peptides, especially regarding macrophage regulation, remain inadequately elucidated.

To overcome these limitations, we designed a novel peptide by incorporating histidine substitutions into MLT. While histidine substitution is established in anticancer and cell-penetrating peptides, its role in anti-inflammatory peptide design is underexplored, particularly for SAP treatment. Our research shows that HMLT exhibits significantly reduced cytotoxicity and robust anti-inflammatory effects, as demonstrated by cytotoxicity evaluation and cytokine analysis. Mechanistic studies reveal its ability to modulate macrophage oxidative stress and inducible nitric oxide synthase (iNOS) expression. Global metabolomic profiling further illustrates the metabolic reprogramming induced by HMLT. Additionally, HMLT demonstrates therapeutic efficacy in a caerulein/lipopolysaccharides (LPS)-induced SAP mouse model, highlighting its potential as an improved venom-derived anti-inflammatory agent.

## Methods

### Peptides

The peptides MLT (C0322HC160-1) and HMLT (C0322HC160-3) were provided in pure form by GenScript Biotech Co., Ltd. (Nanjing, China). The identity and purity (> 95%) of each peptide were confirmed by reversed-phase high-performance liquid chromatography (HPLC) and electrospray ionization mass spectrometry (ESI-MS) analysis (refer to Supporting Information, Fig. S1-S4).

### Cell Culture

RAW 264.7 cells (STCC20020P, Servicebio Technology Co. Ltd., China) were maintained in DMEM medium enriched with 10% (v/v) FBS (F801-500, Bdbio, China), 100 U/mL penicillin, and 100 mg/mL streptomycin (A200-100, Bdbio, China). The cells were cultured in a humidified incubator with a 95% air and 5% CO_2_ atmosphere.

### Evaluation of RAW264.7 Cell Viability

The toxicity of the peptides on RAW 264.7 cells was assessed using the CCK8 assay. Initially, the cells were seeded into 96-well plates at a concentration of 1 × 10^4^ cells per well, allowing them to adhere completely. Subsequently, the cells were treated with 100 µL of serially diluted peptides at final concentrations ranging from 8 µM to 0.06 µM (prepared via two-fold serial dilution: 8, 4, 2, 1, 0.5, 0.25, 0.13, 0.06 µM) and incubated for 24 h at 37 °C. The plates were then incubated for 3 h at 37 °C after 10 µL of CCK8 reagent (HY-K0301, MCE, China) was added to each well. Lastly, the percentage of live cells was calculated by recording the absorbance at 450 nm via a cell imaging multimode reader (Cytation5, Biotek, USA).

### Lactate Dehydrogenase Leakage Assay

RAW 264.7 cells were plated in 96-well plates at a density of 1 × 10^4^ cells per well for 24 h. After this period, the cells were treated with 100 µL of peptide solution at final concentrations ranging from 1 µM to 0.06 µM (1, 0.5, 0.25, 0.13, 0.06 µM) and incubated for another 24 h at 37 °C. Subsequently, the supernatants were harvested for analysis. The release of lactate dehydrogenase (LDH) was measured with LDH activity assay kit (BC0685, Solarbio, China) by the ultraviolet absorbance at 450 nm. Cells that were not treated with peptides served as the negative control, indicating no leakage (0%). In contrast, cells lysed with 0.1% Triton X-100 (T8200, Solarbio, China) were used as the positive control, representing complete leakage (100%). The percentage of LDH leakage was then calculated relative to the positive control values.

### Calcein AM/PI Staining

RAW 264.7 cells were plated in 12-well plates at a density of 1 × 10^5^ cells per well for 24 h. Following this, they were treated with 1 mL of peptide solution at final concentrations of 0.5 µM and 0.13 µM and incubated for an additional 24 h at 37 °C. The cells treated with 0.1% Triton X-100 served as the positive control, whereas the cells that were left untreated served as the negative control. The culture medium was taken out after the incubation. The cells were then washed twice with phosphate-buffered saline (PBS). Subsequently, 1 mL of a medium containing the vital dyes Calcein AM and propidium iodide (PI) (C2015S, Beyotime, China) was added, then the cells were kept at 37 °C for 30 min. Post staining, the cells were washed twice with PBS to remove any excess dye. Finally, images of the stained cells were captured using the cell imaging system (EVOS M7000, Thermo Fisher, USA).

### Determination of TNF-α Levels by ELISA

RAW 264.7 cells were plated in 96-well plates at a density of 1 × 10^4^ cells per well for 24 h. Subsequently, the cells were co-treated with the peptides (MLT at 0.06 µM and 0.13 µM; HMLT at 0.06 µM, 0.13 µM, and 0.5 µM) and 100 ng/mL lipopolysaccharide (LPS) (60747ES10, Yeasen, China) for another 24 h. Cells without any treatment served as the negative control, while those treated with 100 ng/mL LPS alone for 24 h were the positive control. Following treatment, the supernatants were gathered, and mouse TNF-a ELISA Kit (JL10484, Jonlnbio, China) were used to measure the levels of TNF-α.

### Evaluation of Gene Expression Through qPCR

After 24 h of plating in 6-well plates (3 × 10⁵ cells/well), RAW 264.7 cells were stimulated with 100 ng/mL LPS in the presence or absence of peptides. The peptides were used at concentrations determined to be non-cytotoxic in prior assays (MLT, 0.13 µM; HMLT, 0.5 µM, as determined by the CCK-8 assay), and the treatment lasted for 24 h. The positive control group was cells treated with 100 ng/mL LPS only for 24 h, while the negative control group consisted of cells that were not treated. Using an RNA isolation reagent (R401-01, Vazyme, China), total RNA was isolated from the RAW264.7 cells and diluted to 500 ng/mL. Using the hiScript IV All-in-One Ultra RT SuperMix (R433-01, Vazyme, China), which is appropriate for quantitative polymerase chain reaction (qPCR), the RNA was then reverse transcribed into complementary DNA (cDNA). A SYBR Green qPCR master mix (Q711-02, Vazyme, China), primers, cDNA, and RNase-free water were all components of the qPCR reaction combination. The sequences of the primer pairs used in this study were obtained from Primer Bank (https://pga.mgh.harvard.edu/primerbank/). Specifically, for mouse β-actin, the forward primer is 5’-GGCTGTATTCCCCTCCATCG-3’ and the reverse primer is 5’-CCAGTTGGTAACAATGCCATGT-3’; for mouse TNF-α, the forward primer is 5’-CCCTCACACTCAGATCATCTTCT-3’ and the reverse primer is 5’-GCTACGACGTGGGCTACAG-3’; for mouse IL-6, the forward primer is 5’-TAGTCCTTCCTACCCCAATTTCC-3’ and the reverse primer is 5’-TTGGTCCTTAGCCACTCCTTC-3’; for mouse IL-1β, the forward primer is 5’-GCAACTGTTCCTGAACTCAACT-3’ and the reverse primer is 5’-ATCTTTTGGGGTCCGTCAACT-3’; for mouse NOS2, the forward primer is 5’-GTTCTCAGCCCAACAATACAAGA-3’ and the reverse primer is 5’-GTGGACGGGTCGATGTCAC-3’; for mouse CD86, the forward primer is 5’-TGTTTCCGTGGAGACGCAAG-3’ and the reverse primer is 5’-TTGAGCCTTTGTAAATGGGCA-3’.The thermal cycling parameters were as follows: an initial denaturation at 95 °C for 30 s, followed by 40 cycles of denaturation at 95 °C for 10 s and annealing/extension at 60 °C for 30 s.

### Determination of ROS Levels or Mitochondrial Membrane Potential

Flow cytometry was utilized to assess both the intracellular ROS levels and mitochondrial membrane potentials in RAW 264.7 cells. Initially, the cells were seeded into 6-well plates at a density of 3 × 10^5^ cells per well and allowed to adhere. Subsequently, 2 mL of the peptide (MLT, 0.13 µM; HMLT, 0.5 µM) and 100 ng/mL LPS for 24 h of incubation. Cells that received no treatment served as the negative control. The culture medium was taken out after the incubation. The cells were then washed twice with PBS. Subsequently, 1 mL of a medium containing ROS probe DCFH-DA (S0033S, Beyotime, China) or mitochondrial membrane potential Rho123 (Y268251, Beyotime, China) was added, and the cells were incubated for 30 min at 37 °C. After staining, the cells were washed twice with PBS. The cells were then centrifuged to collect and resuspended in 500 µL of PBS. The intracellular ROS levels were determined by analyzing the fluorescence intensity of DCFH using a flow cytometer (NovoCyte Quanteon, Agilent, USA). The mitochondrial membrane potentials were similarly assessed by measuring the fluorescence intensity of rhodamine 123.

### Determination of NO

RAW 264.7 cells were plated in 96-well plates at a density of 1 × 10^4^ cells per well for 24 h. Then they were treated with the peptide (MLT, 0.13 µM; HMLT, 0.5 µM) and 100 ng/mL LPS for a further 24 h. Untreated cells were used as the negative control and cells treated with 100 ng/mL LPS for 24 h served as the positive control. Supernatants were collected, and the levels of NO were measured using the corresponding NO detection kits (S0021S, Beyotime, China).

### Western Blot Analysis of iNOS

RAW 264.7 cells were plated in 6-well plates at a density of 3 × 10^5^ cells per well for 24 h. Then, 2 mL of media contenting peptide (MLT, 0.13 µM; HMLT, 0.5 µM) as added for another 24 h. Subsequently, the cells were harvested, lysed with RIPA buffer containing protease inhibitor. SDS-PAGE was used to separate proteins (10 µg/samples), which were then moved to PVDF membranes. The membranes were blocked in TBST with 5% BSA for 1 h at room temperature. The blots were incubated with the following antibodies for an entire night at 4 °C: β-actin (1:4000; S0B0005, STARTER, China) and iNOS (1:1000; 13120 S, CST, USA). Before imaging using an 2-channel near-infrared (NIR) fluorescent imager (Odyssey DLx, LICRObio, USA), membranes were treated with dylight 800 4× PEG conjugated Anti-rabbit IgG (H + L) (1:300000; 5151P, CST, USA) for 1.5 h at room temperature and washed with TBST for 3 times.

### Non-targeted Metabolomics

After being seeded into 10 cm^2^ dishes, the cells were either treated with 100 ng/mL LPS alone for 24 h (LPS group) or 0.5 µM HMLT with 100 ng/mL LPS for 24 h (peptide group). The negative control (Control group) was made up of cells that were not treated. 400 µL of the extract containing 0.02 mg/mL L-2-chlorophenylalanine (internal standard) was used to extract metabolic products. Methanol and water (4:1, v/v) were used as a solvent mixture to create the extract. After that, the supernatant was moved to an insert-equipped vial for further examination. To create a quality control (QC) sample, the metabolic products from each sample were combined in an identical volume.

Metabolomic analyses were undertaken by Majorbio Bio-Pharm Technology Co. Ltd. On a Thermo UHPLC-Exploris 240 system (Thermo Fisher, USA) with an ACQUITY HSS T3 column (Waters, USA), LC-MS/MS analysis was performed. 0.1% formic acid in water: acetonitrile (95:5, v/v) and 0.1% formic acid in acetonitrile: isopropanol: water (47.5:47.5, v/v) made up the mobile phases. The column temperature was 40 °C, and the flow rate was 0.40 mL/min. The samples’ mass spectrometry signals were acquired using both positive and negative ion scanning modes. The auxiliary gas heating temperature was 425 °C, the sheath gas flow rate was 60 arb, and the auxiliary gas flow rate was 20 arb. In both positive and negative modes, the ion spray voltage was adjusted at 3400 V and − 3000 V, respectively. The normalized collision energy was 20–40-60 V each cycle. The mass range covered by the Data Dependent Acquisition (DDA) mode of data collection was 70–1050.

Following analysis, a data matrix comprising retention time, mass-to-charge ratio, and peak intensity was produced by importing the LC-MS raw data into the metabolomics processing program Progenesis QI. To get metabolite information, the MS and MS/MS mass spectrometry data were compared to the publicly available Metlin and HMDB databases as well as a self-constructed library. The Majorbio Cloud Platform (https://cloud.majorbio.com) received the processed data matrix and it was uploaded for analysis. During the preliminary data analysis, Principal Component Analysis (PCA) was used for quality control. Group differences were discovered using orthogonal least partial squares discriminant analysis (OPLS-DA). Variable importance (VIP) greater than 1 and a P-value less than 0.05 were used to identify differential metabolites. The Majorbio Cloud Platform was also used for more complex analyses, including heatmap creation and Kyoto Encyclopedia of Genes and Genomes (KEGG) enrichment.

### Animals

All experiments utilized female C57BL/6 mice (6–8 weeks old, 15–18 g), which were purchased from Hangzhou Medical College. Upon arrival, the mice were acclimatized for one week under standard housing conditions (temperature: 20–26 °C, 12 h light/dark cycle) with free access to food and water. Thereafter, the animals were randomly divided into four experimental groups (*n* = 7 per group) prior to model induction. The animal use protocol has been reviewed and approved by the Institutional Animal Care and Use Committee, Zhejiang Center of Laboratory Animals (Approval number: ZJCLA-IACUC-20011016). The SAP model was established following previously described methods [30]. Briefly, the mice received intraperitoneal (IP) injections of caerulein (50 µg/kg; HY-A0190, MCE, USA) in normal saline (NS) every hour for 7 h. Immediately following the last caerulein injection, a single injection of LPS (10 mg/kg; 60747ES10, YEASEN, China) was administered. HMLT (0.1 or 1 mg/kg), MLT (1 mg/kg), or an equivalent volume (200 µL) of normal saline was administered 1 h before and 2 h after the first caerulein injection. 24 h after the first caerulein injection, blood was collected and the mice were euthanized to obtain pancreatic tissues for subsequent experiments.

### Determination of Lipase and Amylase Levels

After allowing the mouse blood to stand at room temperature for 2 h, it was centrifuged at 3000 rpm for 15 min at 4℃ to obtain serum. The levels of lipase and amylase in the serum were then measured according to the instructions provided in the lipase assay kit (A054-2-1, Nanjing Jiancheng Bioengineering Insitute, China) and α-amylase assay Kit (C016-1–2, Nanjing Jiancheng Bioengineering Insitute, China), respectively.

### Quantification of Serum AST, ALT, CREA, and BUN

Serum biochemical parameters were assayed according to standard protocols. Briefly, serum samples stored at −40 °C were thawed on ice. Measurements were performed using an automatic biochemical analyzer (7180, Hitachi, Japan) with corresponding commercial kits (Szybio, China). The analyzer was calibrated, and quality control was conducted following the manufacturer’s instructions. The concentrations of alanine aminotransferase (ALT), aspartate aminotransferase (AST), blood urea nitrogen (BUN), and creatinine (CREA) were determined for all samples.

### Histopathological Analysis and Immunohistochemistry

Fresh pancreas was initially fixed overnight in 4% paraformaldehyde. They were subsequently rinsed in tap water for 2 h, followed by dehydration in an ethanol gradient. Afterward, the tissues were embedded in paraffin and cut into 5-µm sections. These sections underwent dewaxing in xylene, rehydration through an ethanol series, and staining with hematoxylin and eosin (H&E) dyes. For TNF-α staining, endogenous peroxidase activity was blocked with 3% H₂O₂ for 8 min. The sections were then incubated overnight with a diluted anti-TNF-α antibody (1:200; ba0131, boster, China). Subsequently, each section was incubated with an HRP-labeled secondary antibody and all slides were observed and captured under a slide scanning system (SQS-120P, Teksqray, China). For CD206 and PKM2 staining, the sections were incubated overnight with diluted anti-CD206 antibody (1:400; 24595 S, CST, USA) and anti-PKM2 antibody (1:400; 4053 S, CST, USA). Each section was then incubated with proper secondary antibodies. All slides were observed and captured under a digital scanner (pannoramic scan ii, 3DHISTECH, Hungary).

### Statistical Methods

Statistical analysis was performed using GraphPad Prism 8, version 8.2.1 (441) (GraphPad software, Boston, MA, USA). Statistical tests are two-tailed, unpaired Student’s t-tests or an ordinary one-way ANOVA with Dunnett’s multiple comparisons test. All results are reported as means ± standard deviations.

## Results

### HMLT Demonstrates Reduced Cytotoxicity while Preserving Anti-inflammatory Activity in Macrophages

Histidine substitution is an established strategy for reducing the cytotoxicity of cationic amphiphilic peptides [31, 32]; however, its effect on their anti-inflammatory efficacy remains poorly understood. To explore this, we designed a novel peptide, HMLT (GIGAVLHVLTTGLPALISWIHRHRQQ-NH₂), by substituting lysine residues in the parent MLT sequence (GIGAVLKVLTTGLPALISWIKRKRQQ-NH₂). As shown in the Table 1, substitution of three lysine residues with histidine residues in HMLT resulted in only minimal changes in the theoretical isoelectric point (pI) and hydrophilicity, the latter assessed by the grand average of hydropathicity (GRAVY, which increased from 0.27 to 0.35). Both peptides maintained high solubility (> 30 mg/mL in deionized water), demonstrating favorable drug-like properties characteristic of cationic amphipathic peptides. The slight increase in HPLC retention time (17.49 to 18.17 min) further corresponds to the modest change in hydrophobicity.

**Table 1:** Characterization data of MLT and HMLT Peptides

Peptide	Sequence	Mw^a^	pI^b^	GRAVY^c^	H-tR (min)^d^	Solubility (mg/mL)^e^
MLT	GIGAVLKVLTTGLPALISWIKRKRQQ-NH₂	2846.48	pH 12.43	0.27	17.49	> 30.00
HMLT	GIGAVLHVLTTGLPALISWIHRHRQQ-NH₂	2873.38	pH 12.40	0.35	18.17	> 30.00

^a^ Theoretical molecular weight (Mw)

^b^ Theoretical isoelectric point (pI)

^c^ Grand average of hydropathicity (GRAVY). A lower value indicates stronger hydrophilicity of the peptide

^d^ RP-HPLC retention time of peptides (5–65% acetonitrile in mobile phase B over 25 min at a flow rate of 1 mL/min; mobile phase A: 0.065% trifluoroacetic acid in water, mobile phase B: 0.05% trifluoroacetic acid in acetonitrile)

^e^ Solubility in deionized water, dissolved by sonication

**Fig. 1:**
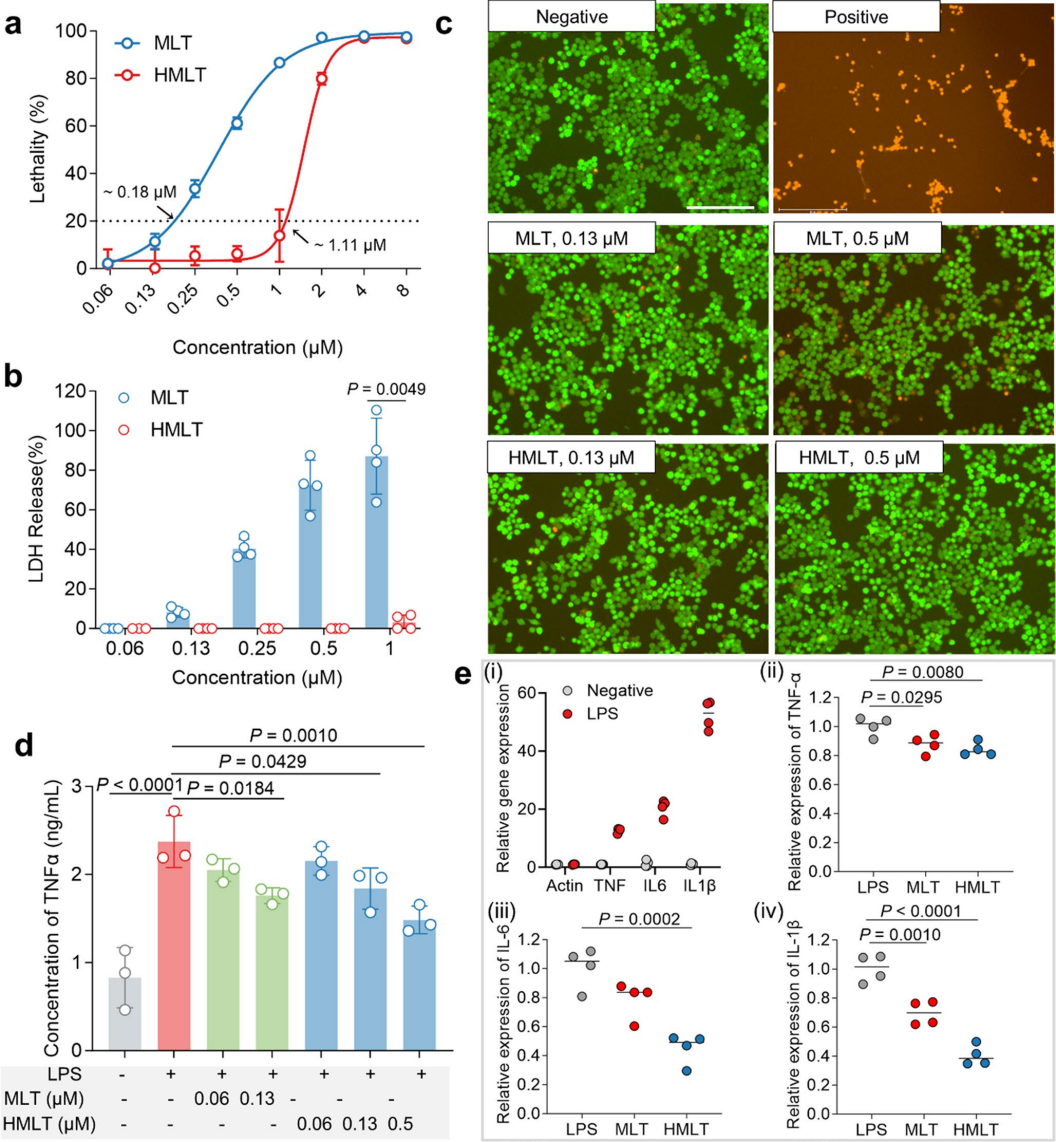
Impact of histidine substitution on the cytotoxicity and anti-inflammatory activity of MLT in RAW 264.7 macrophages.** a** Cytotoxicity evaluation of MLT and HMLT in RAW 264.7 macrophages using the CCK-8 assay. Cells were treated with various concentrations of peptides for 24 h at 37 °C, followed by incubation with CCK-8 reagent for an additional 3 h. **b** LDH release assay. Cells were treated with different concentrations of peptides for 24 h at 37 °C. Supernatants were collected to measure LDH activity, with untreated cells representing 0% release and cells lysed with 0.1% Triton X-100 representing 100% release. Differences between the MLT and HMLT groups were assessed using Student’s t-test. **c** Live/dead staining with calcein-AM and propidium iodide (PI). After 24 h of peptide treatment, cells were stained with calcein-AM/PI for 30 min and imaged using an EVOS M7000 microscope. Live cells are stained green (calcein); dead cells are stained red (PI). Scale bar: 125 μm. **d** TNF-α levels in cell supernatants measured by ELISA following 24 h of treatment. Statistical differences were analyzed by one-way ANOVA with Dunnett’s multiple comparisons test. **e** Relative mRNA expression of inflammatory factors detected by RT-qPCR. Cells were treated for 24 h with 100 ng/mL LPS alone or in combination with peptide. i, Comparison of TNF-α, IL6, and IL1β expression between the negative control and LPS-only groups; ii–iv, Relative expression of TNF-α, IL6, and IL1β across the LPS, MLT, and HMLT groups, respectively. Differences between the negative control and LPS groups were analyzed using Student’s t-test. Comparisons among the LPS and peptide-treated groups were performed using one-way ANOVA with Dunnett’s multiple comparisons test. MLT and HMLT were used at their respective safe concentrations (0.13 µM and 0.5 µM) for functional comparison

Driven by the need for safer therapeutics, this study aimed to develop a novel anti-inflammatory peptide with reduced cytotoxicity for SAP treatment, based on the strategy of modulating macrophage function. Consequently, macrophages are the intended direct cellular targets of HMLT. Assessing the therapeutic window, defined as the disparity between the effective anti-inflammatory dose and the cytotoxic dose, on this target cell type is the most relevant metric for validating its safety and potential applicability. Therefore, we first evaluated the cytotoxicity of HMLT in RAW 264.7 macrophages, a canonical model for studying macrophage polarization and inflammatory responses, using a CCK-8 assay. As shown in Fig. 1a, MLT exhibited potent cytotoxicity against RAW264.7 cells, with a 20%-lethal concentration (LC20) of 0.18 µM. In contrast, HMLT had a significantly higher LC20 of 1.11 µM, indicating reduced cytotoxicity. This finding was corroborated by LDH release experiments, where MLT showed LDH release at 0.13 µM, while HMLT required a concentration of 1 µM for detection (Fig. 1b). Calcein-AM/PI staining at 0.5 µM further confirmed that HMLT induced minimal cell death compared to MLT (Fig. 1c).

To further evaluate the potential off-target cytotoxicity of the peptides in the context of SAP, a condition involving pancreatic acinar cell injury, we also assessed their effects on mouse acinar 266-6 cells using the CCK-8 assay. This step is critical for understanding whether the peptides might exacerbate pancreatic damage while targeting macrophages. Notably, HMLT also exhibited a higher cytotoxicity threshold in acinar cells compared to MLT. The LC₂₀ values for MLT and HMLT in 266-6 cells were about 0.32 µM and 1.04 µM, respectively (Fig. S5), further supporting its improved safety profile across relevant cell types.

Given that TNF-α, IL-6, and IL-1β are pivotal upstream mediators driving the “cytokine storm” and organ injury in SAP, we next assessed whether HMLT retained anti-inflammatory function. TNF-α, a key inflammatory mediator, was measured via ELISA. Both MLT and HMLT suppressed LPS-induced TNF-α release in a concentration-dependent manner, with significant inhibition observed at 0.13 µM. Owing to its reduced cytotoxicity, HMLT could be administered at higher concentrations, such as 0.5 µM and even 1 µM, whereas the application of MLT was limited by its toxicity profile and could not be further increased. RT-qPCR analysis revealed that HMLT more effectively reduced the mRNA expression of TNF-α, IL-6, and IL-1β compared to MLT at safe concentrations (Fig. 1e). These results suggest that the expanded therapeutic window enables stronger anti-inflammatory efficacy with only a moderate increase in concentration, without introducing additional cytotoxicity, which underscores the fundamental advantage of HMLT.

### HMLT Attenuates Intracellular ROS and Mitigates LPS-Induced Mitochondrial Dysfunction

**Fig. 2:**
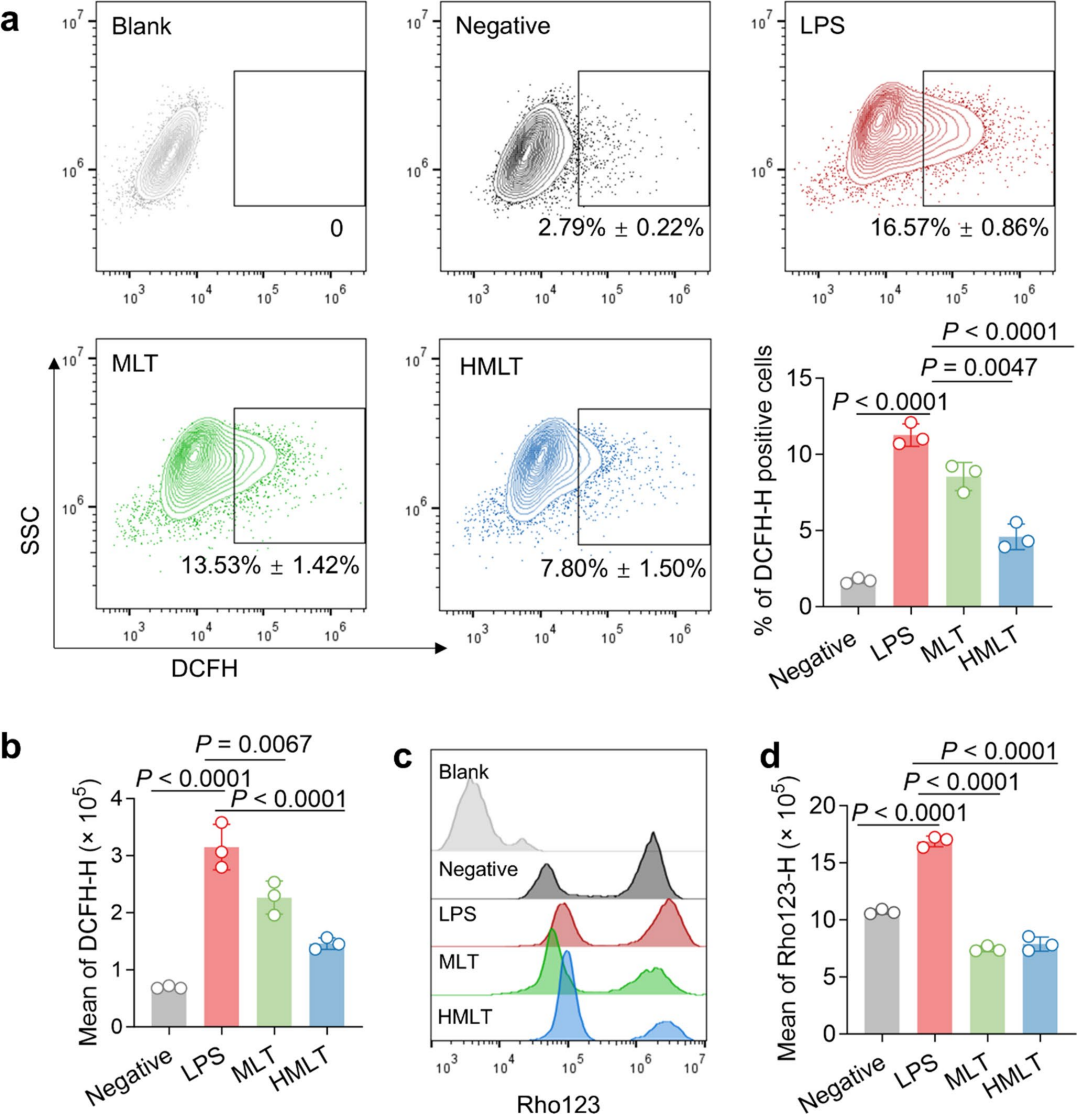
Effects of MLT or HMLT on intracellular ROS and mitochondrial membrane potential in RAW 264.7 macrophages.** a** Flow cytometry analysis of intracellular ROS after cells were treated with LPS or LPS combined with peptide for 24 h, using DCFH as the ROS fluorescent probe. The proportion of DCFH-positive cells is shown as mean ± SD from three independent experiments. **b** Average fluorescence intensity of intracellular ROS after different treatments, expressed as the mean of DCFH-H, with data normalized by subtracting the value of the blank group (cells without probe incubation). Differences between the LPS and other groups were analyzed using one-way ANOVA with Dunnett’s multiple comparisons test. **c** Flow cytometry analysis of mitochondrial membrane potential in cells after different treatments, using Rho123 as the mitochondrial membrane potential probe. **d** Average fluorescence intensity of mitochondrial membrane potential after different treatments, expressed as the mean of Rh123-H, with data normalized by subtracting the value of the blank group. Differences between the LPS and other groups were analyzed using one-way ANOVA with Dunnett’s multiple comparisons test. MLT and HMLT were used at their respective safe concentrations (0.13 µM and 0.5 µM) for functional comparison

Oxidative stress, driven by ROS accumulation, plays a pivotal role in inflammatory signaling [15, 16]. Using the fluorescent probe DCFH-DA, we found that LPS increased ROS-positive cells from 2.79% to 16.57%. Treatment with MLT (0.13 µM) and HMLT (0.5 µM) reduced this proportion to 13.53% and 7.8%, respectively (Fig. 2a). Mean fluorescence intensity analysis confirmed that HMLT more effectively suppressed ROS levels, reducing fluorescence to 46.34% of that in LPS-treated cells, compared to 71.57% with MLT (Fig. 2b).

Given the interplay between ROS and mitochondrial integrity [33, 34], we evaluated mitochondrial membrane potential (ΔΨm) using rhodamine 123 (Rho123) staining. As shown in Fig. 2c and d, compared with the negative group, LPS induced an increase in the average fluorescence intensity of Rho123, indicating that ΔΨm was disrupted at this time. When treated with MLT, compared with the LPS group, the average fluorescence intensity was significantly reduced, indicating that MLT could protect mitochondria and reduce the damage caused by oxidative stress. The treatment with HMLT also yielded the same result, indicating that even after the substitution of histidine, this protective effect can still be maintained. These findings demonstrate that HMLT effectively reduces oxidative stress and preserves mitochondrial function.

### HMLT Suppresses iNOS Expression and NO Production

**Fig. 3:**
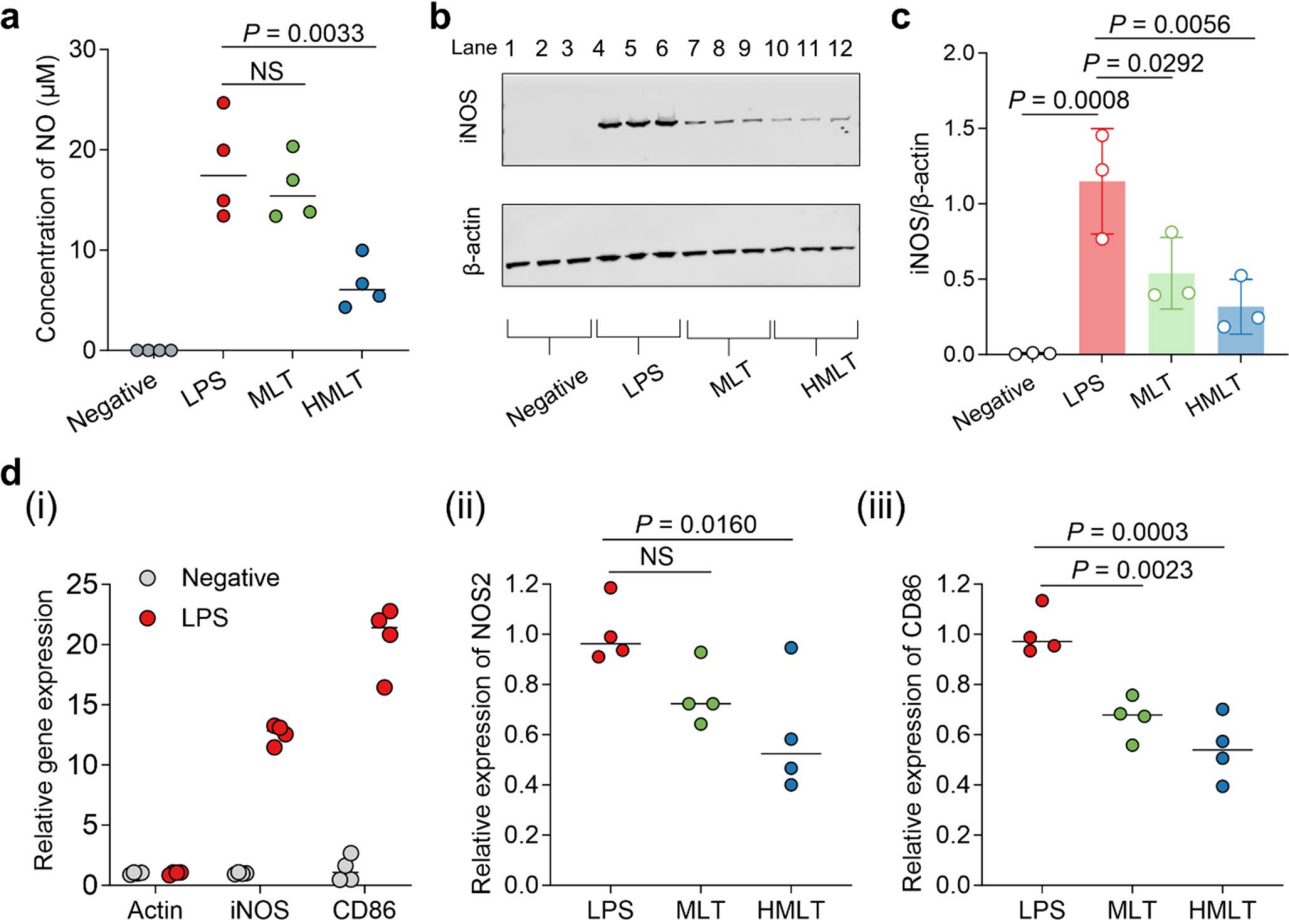
Effects of MLT or HMLT on NO production or related indicators in RAW 264.7 macrophages.** a** Detection of NO. Cells were treated with LPS or LPS combined with peptide for 24 h at 37℃. The cell supernatants were then collected to measure NO production. No mark on panel if data cannot be detected. **b** Representative Western blots showing iNOS protein expression in cells after 24 h of different treatments; **c** Quantitative analysis of iNOS expression from three independent experiments, performed using ImageJ software for grayscale densitometry. **d** QPCR detection of relative expression of iNOS (NOS2) or CD86 in cells treated with LPS or LPS combined with peptide for 24 h. Differences between the negative control and LPS groups were analyzed using Student’s t-test. Differences between the LPS and other groups were analyzed using one-way ANOVA with Dunnett’s multiple comparisons test. MLT and HMLT were used at their respective safe concentrations (0.13 µM and 0.5 µM) for functional comparison

RNS, particularly nitric oxide (NO), contribute significantly to inflammatory damage [35, 36]. Since NO readily diffuses across cell membranes, we measured its accumulation in the cell supernatant. Figure 3a shows that NO was undetectable in untreated cells but significantly elevated in LPS-stimulated macrophages. Treatment with MLT and HMLT reduced NO levels to 63.82% and 36.18% of those in the LPS group, respectively. This reduction suggests that HMLT may exert anti-inflammatory effects via the iNOS pathway, as NO generation is closely linked to iNOS activity. Western blot analysis confirmed that both peptides significantly reduced iNOS protein expression (Fig. 3b and c, Fig. S6). Quantitative PCR further supported this finding, showing that LPS increased NOS2 (iNOS) gene expression by over 190-fold, while HMLT treatment reduced it to approximately 60% of LPS-induced levels (Fig. 3d). These results underscore the importance of iNOS inhibition in HMLT’s anti-inflammatory action.

Additionally, we assessed CD86 expression, a marker of pro-inflammatory M1 macrophages. Co-incubation with 0.5 µM HMLT significantly reduced CD86 expression to nearly half of that in the LPS-treated group (Fig. 3d), suggesting that HMLT may attenuate M1 polarization, further corroborating its anti-inflammatory properties. Overall, the suppression of NO production represents a key mechanism underlying HMLT’s anti-inflammatory effects.

#### HMLT Alleviates LPS-Induced Metabolic Dysregulation in Macrophages

**Fig. 4:**
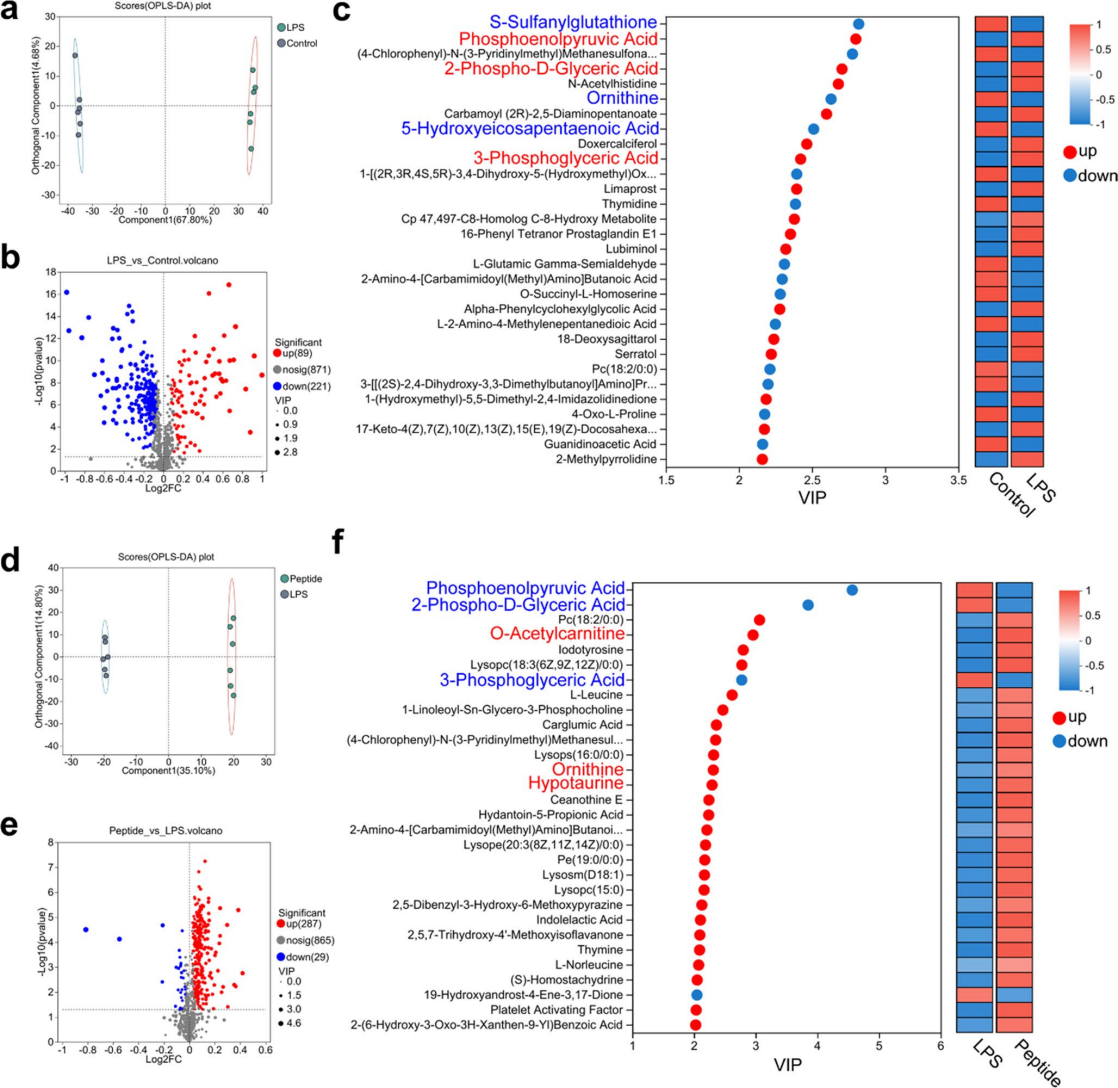
Effects of HMLT on the metabolism of LPS-induced RAW 264.7 macrophages.** a** OPLS-DA results of metabolites between the control group (untreated) and the LPS group (treated with LPS only). **b** Volcano map of differential metabolites between the control and LPS groups. The horizontal axis represents the fold change in metabolite expression differences between the two groups, while the vertical axis represents the statistical significance of these differences. **c** Expression profile and VIP of metabolites for comparison between the control and LPS group. **d** OPLS-DA results of metabolites between the LPS group and the peptide group (LPS combined with 0.5 µM HMLT). **e** Volcano map of differential metabolites between the LPS and peptide groups. The horizontal axis represents the fold change in metabolite expression differences between the two groups, while the vertical axis represents the statistical significance of these differences. **f** Expression profile and VIP of metabolites for comparison between the LPS and peptide group

Comprehensive non-targeted metabolomic analysis revealed the metabolite composition and metabolic pathways affected by LPS alone or in combination with HMLT. We detected 684 metabolites in positive ion mode and 497 in negative ion mode (Tab. S1). Principal component analysis (PCA) showed clear separation among experimental groups and quality control samples, particularly between the LPS and control groups (Fig. S8). Orthogonal partial least squares discriminant analysis (OPLS-DA) further confirmed distinct metabolic profiles between the control and LPS groups (Fig. 4a). A total of 310 metabolites were significantly altered (VIP > 1, *P* < 0.05) in response to LPS, with 89 upregulated and 221 downregulated (Fig. 4b, Tab. S2). Among the top 30 differentially abundant metabolites, the endogenous antioxidants sulfanylglutathione, ornithine, and 5-hydroxyeicosapentaenoic acid were downregulated, while key glycolytic intermediates, phosphoenolpyruvate (PEP), 2-phospho-D-glyceric acid, and 3-phosphoglyceric acid, were upregulated (Fig. 4c).

OPLS-DA also revealed a clear separation between the peptide group and LPS group (Fig. 4d). In this comparison, 316 metabolites were significantly altered (VIP > 1, *P* < 0.05), with 287 upregulated and 29 downregulated (Fig. 4e, Tab. S3). Among the top 30 differentially abundant metabolites, endogenous antioxidants including O-acetylcarnitine, ornithine, and hypotaurine were downregulated. Notably, PEP, 2-phospho-D-glyceric acid, and 3-phosphoglyceric acid remained elevated (Fig. 4f). These findings indicate that HMLT alleviates LPS-induced metabolic disturbances by enhancing endogenous antioxidant levels and modulating glycolytic intermediates.

**Fig. 5:**
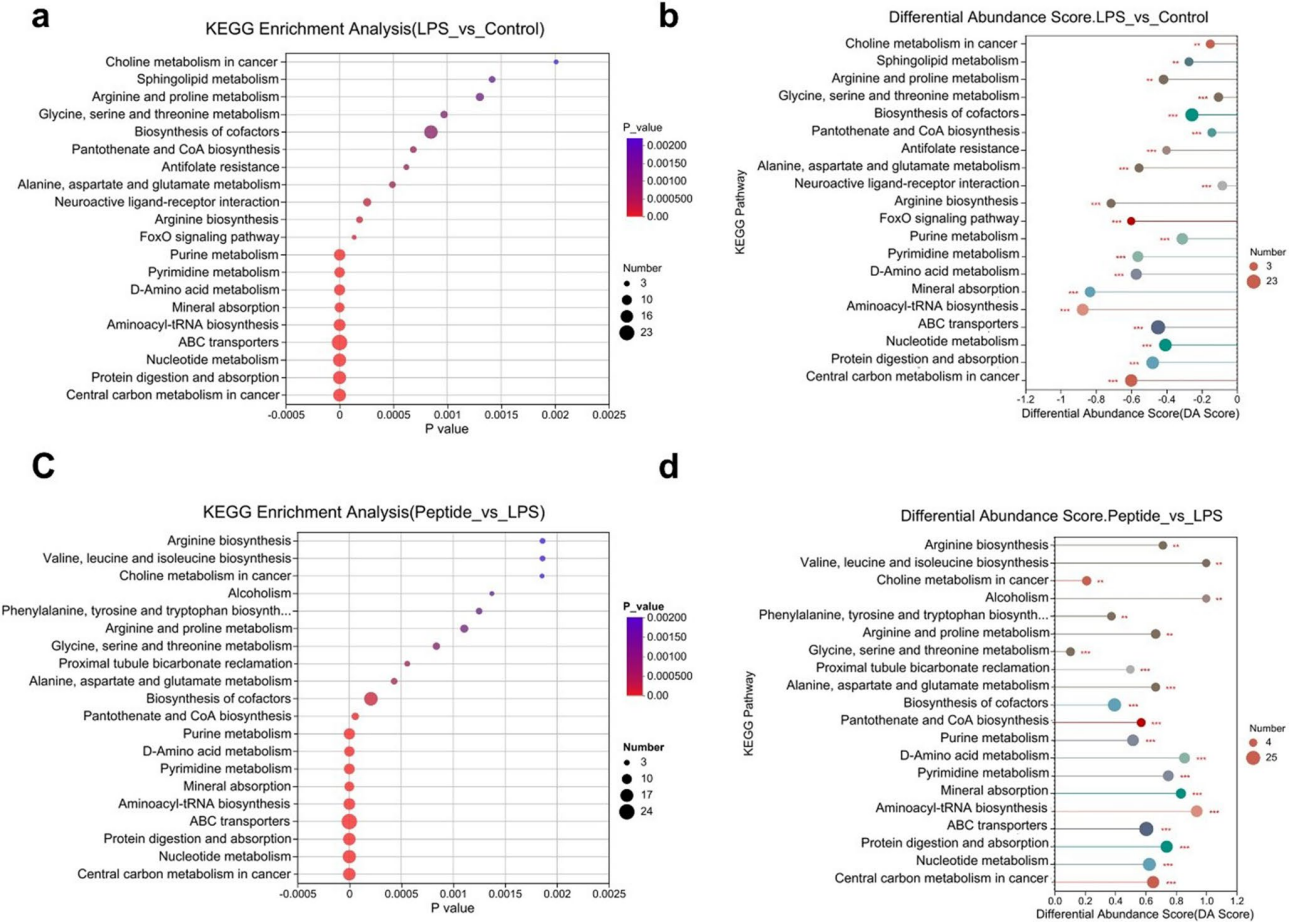
The KEGG enrichment analysis of differential metabolites.** a** The KEGG enrichment analysis of differential metabolites between the control (no treated) and LPS groups (LPS treated only). The horizontal axis represents the enrichment significance value, and the vertical axis represents KEGG pathways. The size of the bubbles in the panel indicates the number of compounds enriched in the pathway. **b** The differential abundance score analysis of metabolites between the control and LPS groups. The horizontal axis represents the differential abundance score, and the vertical axis represents the names of KEGG pathways. The size of the dots indicates the number of annotated differential metabolites in the pathway. Dots distributed to the right of the 0-axis line indicate that the pathway is generally upregulated, while dots to the left indicate that the pathway is generally downregulated. **c** The KEGG enrichment analysis of differential metabolites between the LPS and peptide groups (LPS combined with 0.5 µM HMLT). **d** The differential abundance score analysis of metabolites between the LPS and peptide groups

KEGG pathway analysis comparing the LPS and control groups revealed significant enrichment in central carbon metabolism, protein digestion and absorption, nucleotide metabolism, ABC transporters, aminoacyl-tRNA biosynthesis, mineral absorption, D-amino acid metabolism, purine metabolism, and pyrimidine metabolism (Fig. 5a). The DA Score indicated a general downregulation trend across these pathways (Fig. 5b). Similar pathways were enriched when comparing the peptide and LPS groups, though the DA Score suggested that HMLT treatment tended to reverse LPS-induced alterations, restoring metabolic homeostasis (Fig. 5c and d).

### HMLT Ameliorates Experimental Severe Acute Pancreatitis

**Fig. 6:**
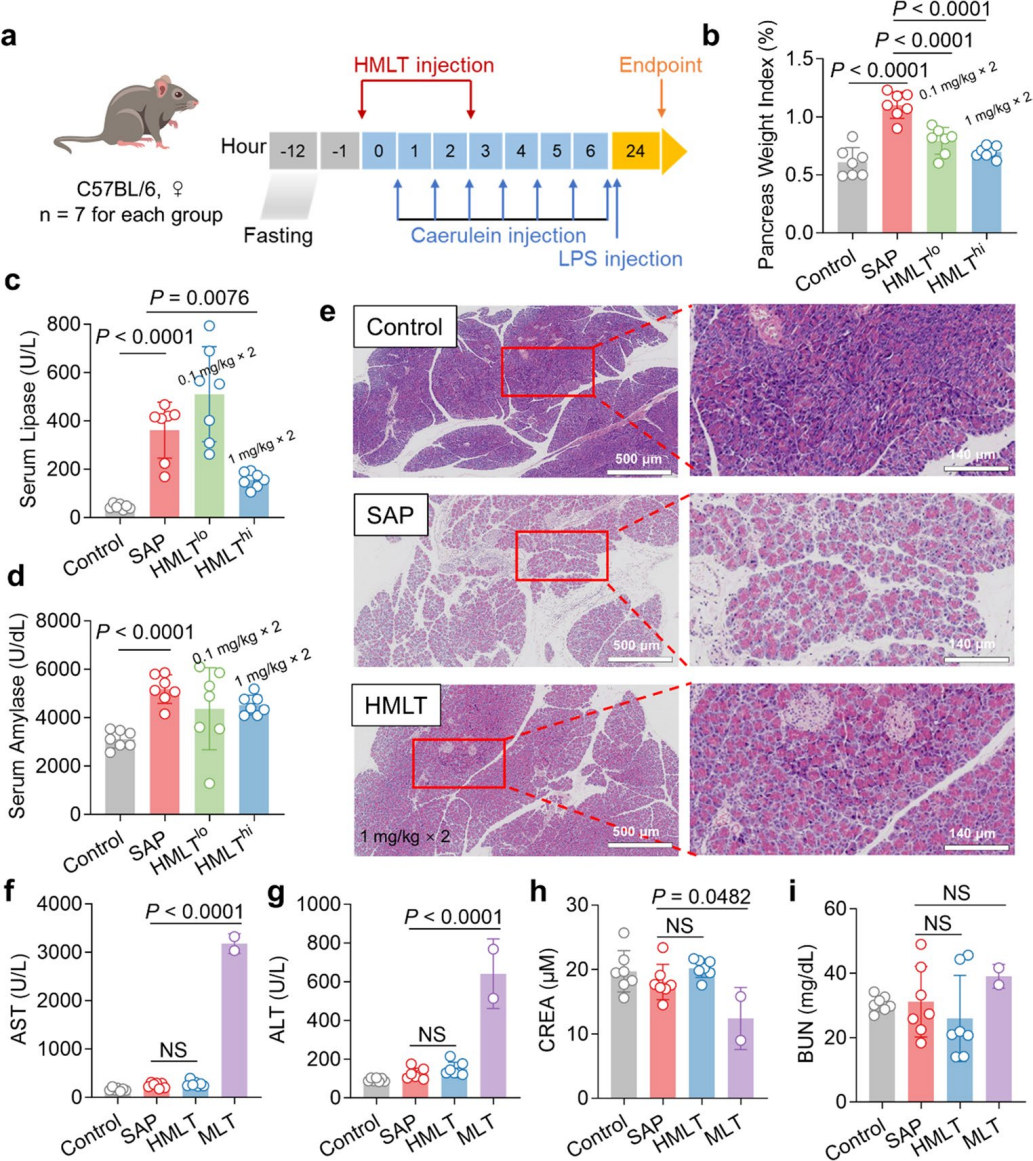
The ameliorative effect of HMLT on SAP.** a** Schematic diagram of the construction of the SAP model and the experimental process. For the SAP model, the mice received intraperitoneal injections of caerulein (50 µg/kg) in normal saline every hour for 7 h. Immediately following the last caerulein injection, a single injection of LPS (10 mg/kg) was administered. HMLT (0.1 mg/kg or 1 mg/kg body weight) or an equivalent volume of normal saline was administered 1 h before and 2 h after the first caerulein injection. Control, healthy mice; SAP, model mice; HMLT, mice treated with two intraperitoneal injections of HMLT. **b** Bar plots present the quantification of pancreas weight index in in different groups (*n* = 7). **c**,** d** Bar plots present the quantification of serum lipase and amylase in different groups (*n* = 7). **e** Representative images display pancreatic H&E staining; scale bar, 500 μm. The images on the right show the magnified view of the selected area on the left; scale bar, 140 μm. Differences between the SAP and other groups were analyzed using one-way ANOVA with Dunnett’s multiple comparisons test for the above panels. **f-i** Serum levels of AST, ALT, CREA and BUN (*n* = 7 per group, except for the MLT group in which only 2 mice survived to endpoint)

SAP involves uncontrolled local inflammation progressing to SIRS, leading to multi-organ dysfunction and life-threatening complications. In this study, we established a murine model of SAP to evaluate the therapeutic potential of HMLT. The HMLT dosage was selected based on the concentration range previously established for native MLT in related inflammatory models [37]. This initial range was refined through a pilot experiment, in which groups of three mice received HMLT at 0.1, 1, and 2 mg/kg (Fig. S7). The pilot results demonstrated a therapeutic effect that was dose-dependent between 0.1 and 1 mg/kg, leading to the selection of these two doses for the main therapeutic evaluation in this study. The experimental timeline is summarized in Fig. 6a. As shown in Fig. 6b-6d, levels of lipase, amylase, and the pancreatic weight index were assessed 24 h after the initial caerulein injection. All three parameters were significantly elevated in the SAP group compared to normal controls, indicating pancreatic edema and loss of exocrine function. Even at a low dose of 0.1 mg/kg body weight, HMLT was able to reduce the pancreas-to-body weight index and serum amylase levels compared to the SAP group. When the dosage was increased to 1 mg/kg, HMLT significantly lowered these indicators, demonstrating its protective effects on pancreatic structure and exocrine function. Histopathological examination of pancreatic tissue (Fig. 6e) further revealed diffuse and localized edema, interstitial expansion, inflammatory infiltration by neutrophils and lymphocytes, as well as hemorrhage and necrosis in SAP mice, consistent with characteristic pancreatic pathology. These changes were markedly attenuated in HMLT-treated mice, which showed reduced edema and diminished inflammatory cell infiltration. Collectively, these results indicate that HMLT effectively mitigates pathological damage associated with SAP.

In a parallel experiment, the safety profiles of HMLT and native MLT were compared in the same SAP model. Both peptides were administered intraperitoneally at 1 mg/kg (two doses in total). Mice receiving MLT exhibited rapid clinical deterioration after the second dose, with only 2 out of 7 surviving to the experimental endpoint (the remaining subjects either died or were euthanized in accordance with ethical guidelines). Serum biochemical analysis indicated that MLT administration significantly elevated AST and ALT levels, reflecting notable liver injury. In contrast, HMLT treatment did not induce marked alterations in these hepatic markers or in renal function parameters, including CREA and BUN (Fig. 6f-6i). These results underscore the substantially improved safety profile of HMLT relative to the native peptide.

### HMLT Attenuates Inflammation and Reprograms Glycolytic Activity in Macrophages

**Fig. 7:**
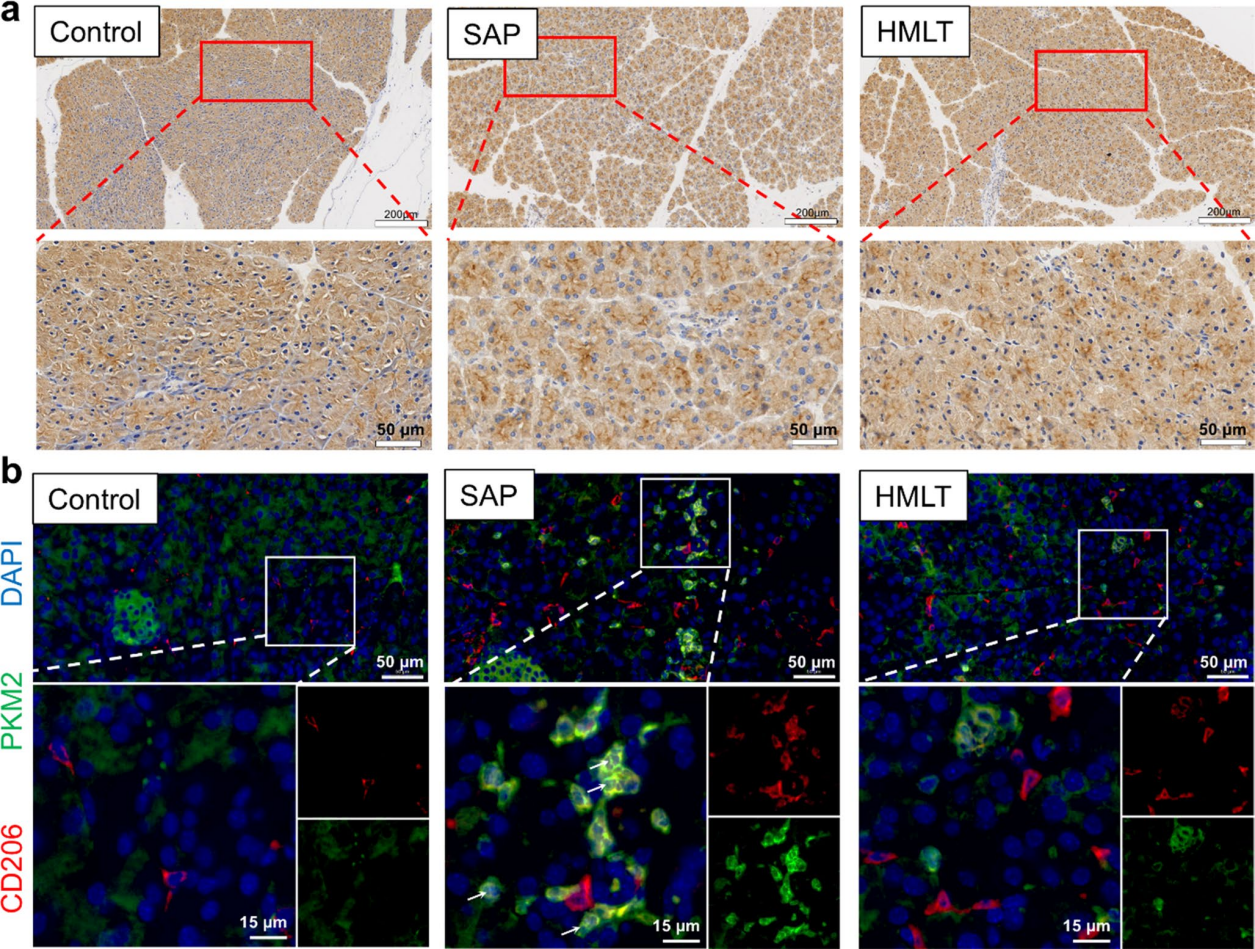
Immunostaining analysis of pancreatic tissue.** a** Representative images show pancreatic immunohistochemical staining of TNF-α; scale bar, 200 μm. The images below display the magnified view of the selected area; scale bar, 50 μm. **b** Representative images show pancreatic immunofluorescence staining of CD206 (red) and PKM2 (green); nuclei are stained with DAPI (blue); scale bar, 50 μm. The images below display the enlargement of the selected area in the merged figure (scale bar, 15 μm), as well as the separate channel images. White arrows indicate the nuclear PKM2 signal

We further evaluated the expression of inflammatory cytokines in pancreatic tissue across the different treatment groups. Immunohistochemical staining for TNF-α (Fig. 7a) revealed that caerulein/LPS challenge markedly induced TNF-α expression in pancreatic tissue compared to healthy controls, indicating a highly inflammatory state. In contrast, HMLT treatment significantly suppressed caerulein/LPS-induced TNF-α production, underscoring its potent anti-inflammatory activity in vivo.

Macrophage infiltration and metabolic status were evaluated by immunofluorescence using CD206 (an M2 macrophage marker) and PKM2 (a key glycolytic enzyme). In control mice, CD206^+^ cells were sparse, and PKM2 expression was largely confined to pancreatic islet cells. In the SAP model, which shares a pro-inflammatory environment similar to LPS stimulation in vitro, infiltrating CD206^+^ M2 macrophages showed markedly increased PKM2 expression along with prominent nuclear translocation (indicated by white arrows in Fig. 7b). This translocation is mechanistically linked to a shift in PKM2 from its active, tetrameric state toward dimeric/monomeric forms that favor nuclear entry and transcriptional activity. This co‑expression profile suggests a hyper‑glycolytic state that likely compromises their anti‑inflammatory function. Treatment with HMLT significantly suppressed both the overall upregulation of PKM2 and its nuclear accumulation, suggesting a potential stabilization of its tetrameric, metabolically active form. These coordinated changes indicate a functional reduction in glycolytic activity within M2 macrophages. Such a metabolic shift from glycolysis toward oxidative phosphorylation (OXPHOS) is linked to enhanced anti‑inflammatory capacity. Together, these in vivo results demonstrate that HMLT reprograms macrophage metabolism and helps restore inflammatory balance.

### Proposed Mechanism of HMLT Action

**Fig. 8:**
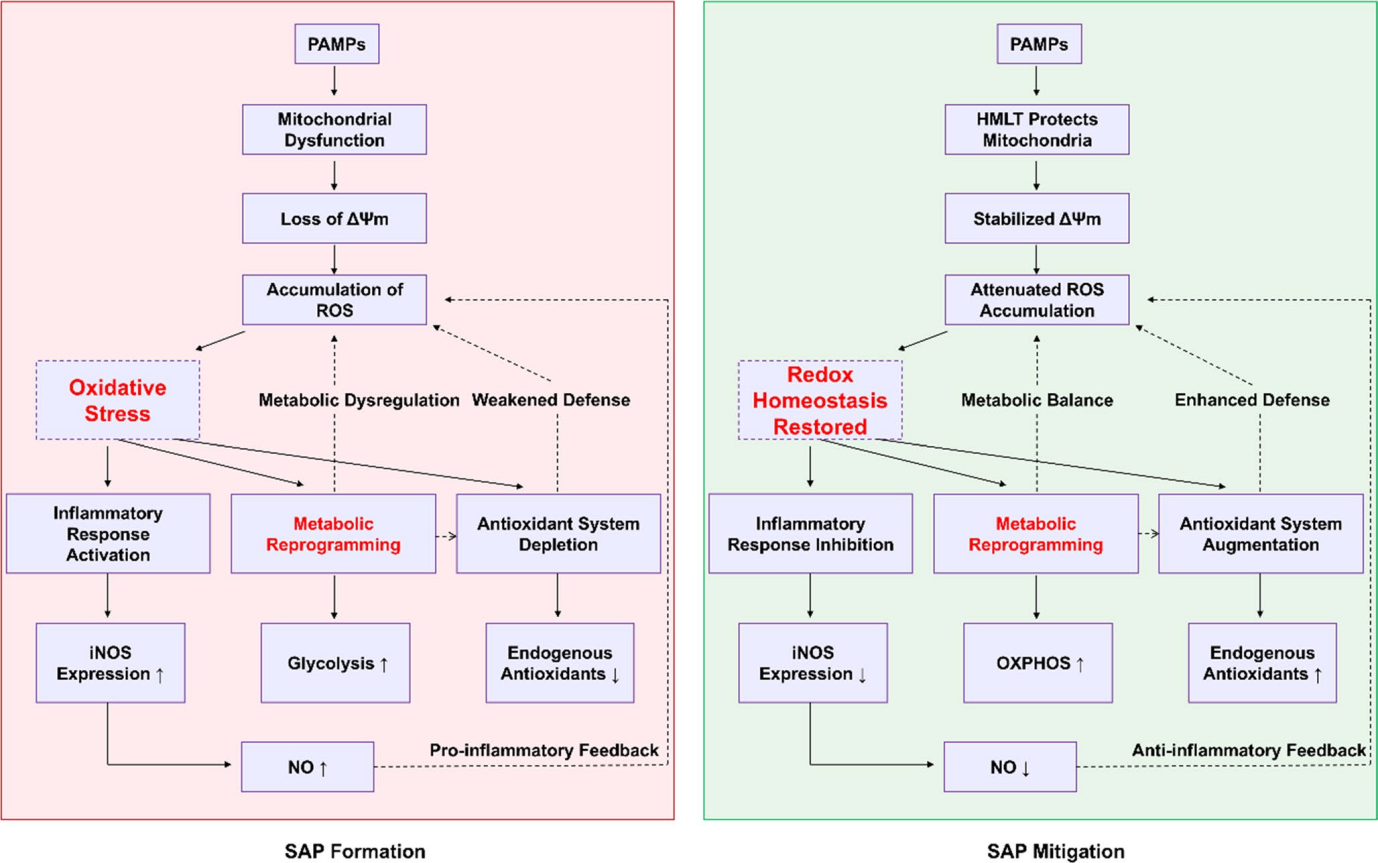
Proposed Mechanism of HMLT Action. During SAP, PAMPs (e.g., LPS) induce mitochondrial dysfunction in macrophages, resulting in loss of ΔΨm, ROS accumulation, oxidative stress, and amplified inflammation (increased iNOS/NO). Glycolysis is enhanced, exacerbating redox imbalance. HMLT treatment preserves mitochondrial integrity, stabilizes ΔΨm, reduces ROS, and suppresses inflammation (decreased iNOS/NO). It shifts metabolism from glycolysis to OXPHOS and enhances antioxidant metabolites, collectively restoring redox homeostasis and attenuating SAP progression

In this study, we developed a novel peptide, designated HMLT, through a histidine-substitution strategy. HMLT exhibits low toxicity and potent anti-inflammatory properties, primarily mediated via the restoration of redox homeostasis. The mechanism can be summarized as follows (Fig. 8): during SAP formation, activation by pathogen-associated molecular patterns (PAMPs), including LPS, induces mitochondrial dysfunction in macrophages, leading to loss of ΔΨm and substantial ROS accumulation, which culminates in severe oxidative stress. This oxidative burden amplifies the inflammatory response, marked by upregulation of iNOS expression and enhanced NO release. Concurrently, PAMP stimulation enhances glycolytic activity in macrophages. The combined impact of ROS overproduction and depletion of endogenous antioxidants exhausts cellular antioxidant defenses, establishing a self-propagating cycle that aggravates oxidative damage.

In contrast, HMLT treatment counteracts these pathological alterations through multiple mechanisms. It preserves mitochondrial integrity, stabilizes ΔΨm, and markedly attenuates intracellular ROS accumulation induced by LPS and related stimuli. Consequently, HMLT facilitates rebalancing of the cellular redox state. This is accompanied by suppression of inflammatory signaling, demonstrated by reduced iNOS expression and decreased NO production. Furthermore, HMLT reprograms macrophage metabolism by shifting the balance from glycolysis toward OXPHOS. Additionally, HMLT enhances the endogenous antioxidant system, elevating levels of metabolites such as acetylcarnitine, ornithine, and hypotaurine. Together, these pleiotropic actions synergistically restore redox homeostasis, ultimately mitigating SAP progression.

## Discussion

Previous studies have shed light on the vital role of histidine in peptide design, especially in the realms of cancer therapy and drug delivery. For instance, Makovitzki et al. found that histidine-rich, host defense-like lytic peptides can effectively suppress solid tumor growth in mice through both intratumoral and systemic inoculation, while exhibiting lower systemic toxicity [31]. Moreover, Zhao et al. showed that engineered histidine-rich peptides enhance endosomal escape, thereby facilitating the antibody-targeted intracellular delivery of functional proteins [32]. These findings highlight the potential of histidine to improve the efficacy and specificity of therapeutic peptides.

Building on this rationale, we applied histidine substitution as a strategy to modulate inflammation. Our research indicates that HMLT, a peptide obtained through histidine substitution, significantly reduces cytotoxicity to macrophages while retaining its anti-inflammatory activity. This is particularly advantageous as it allows for a higher permissible concentration of action, potentially leading to enhanced anti-inflammatory effects. This approach not only mitigates side effects on normal tissues but also optimizes the therapeutic window for treating inflammatory conditions. As a cationic amphipathic peptide, the biological activity of melittin and its derivatives is highly dependent on the formation of its α-helical structure and its membrane-interaction capability [38]. The design of HMLT was guided by structure-based optimization, informed by our prior work on the essential α-helical structure and membrane-interaction capability of cationic amphipathic peptides [39, 40]. This rational approach aimed to preserve the core bioactive conformation while improving pharmacological properties.

The clinical translation of peptide therapeutics, however, is often hampered by poor in vivo stability and short half-life. Common strategies to address these limitations include cyclization, D-amino acid substitution, and PEGylation [41, 42]. Particularly relevant to the pathological milieu of SAP is the massive release of pancreatic enzymes. Notably, lysine residues in the native melittin sequence represent potential cleavage sites for trypsin. Consequently, the histidine substitutions in HMLT may confer a dual advantage: reducing intrinsic peptide toxicity and potentially enhancing resistance to proteolytic degradation in the enzyme-rich SAP environment, thereby indirectly improving local stability and bioavailability. A detailed assessment of pharmacokinetic parameters such as solubility, stability, and half-life will be a crucial focus for subsequent translational development.

Anti-inflammatory activity is closely related to antioxidant capacity [15, 16, 43–45]. Our research indicates that HMLT, like MLT, possesses the ability to reduce the accumulation of intracellular ROS and RNS. From the fluorescence intensity of ROS probes and the release of NO, HMLT even shows better antioxidant effects than MLT. This may be directly related to the introduction of histidine residues. In terms of antioxidant properties, dipeptides containing imidazole groups have been reported to have antioxidant activity due to their metal chelating and free radical scavenging capabilities [46]. We suspected whether HMLT, after histidine substitution, might have similar functions. Our results revealed that within a low concentration range, HMLT indeed demonstrated superior DPPH radical scavenging ability, which may also be related to its excellent anti-inflammatory activity (Fig. S9). Furthermore, transcriptomic data also revealed that HMLT treatment can upregulate endogenous antioxidants. This mechanism has been reported in previous studies, such as alpha-lipoic acid (ALA) [47, 48]. ALA is a potent antioxidant that operates both intracellularly and extracellularly, capable of neutralizing a variety of ROS. Studies have shown that ALA can activate endogenous antioxidants, such as such as vitamin C, vitamin E, or antioxidant enzymes glutathione (GSH), enhance their antioxidant activity, and further reduce oxidative damage.

Regarding the mechanism of action, traditional research suggests that melittin may exert anti-inflammatory effects through pathways such as acting on TLR4/TLR2 receptors or inhibiting the IKK/NF-κB pathway [49–52]. There are also reports that its depolarizing effect on mitochondrial membranes can induce apoptosis in tumor environments. However, in the context of inflammatory diseases, the anti-inflammatory mechanism of melittin derivatives may be more complex and not primarily dependent on mitochondrial membrane interactions. We also discovered that HMLT can significantly downregulate the expression of iNOS, a marker of M1-type macrophages [11, 21]. The polarization of macrophages is closely related to their metabolic state, particularly the glycolytic and OXPHOS process. LPS treatment can activate the glycolytic process in macrophages, which is associated with the polarization state of M1-type macrophages [53, 54]. Under LPS stimulation, M1-polarized macrophages exhibit a Warburg effect similar to that of cancer cells, preferring glycolysis as the primary energy supply [55, 56]. This metabolic shift provides rapid energy supply for macrophages and supplies the necessary substrates for the massive production of inflammatory factors. The metabolites Phosphoenolpyruvic acid, 2-phosphoglyceric acid, and 3-phosphoglyceric acid are key intermediates in the glycolytic pathway. Metabolomics data revealed that these metabolites were significantly elevated under LPS treatment, indicating enhanced glycolysis. Conversely, in the presence of HMLT, these metabolites were significantly downregulated compared to the LPS-treated group, suggesting attenuated glycolysis. The anti-inflammatory role of the key glycolytic enzyme PKM2 relies not only on changes in its expression level but, more crucially, on the intrinsic mechanism of dynamic transition between its tetrameric (high activity) and dimeric (low activity) forms, which regulates metabolic flux and gene expression [57, 58]. HMLT’s inhibition of glycolysis may involve intervention in this transition process. Future work will therefore focus on delineating the underlying mechanism, including direct binding assays, analysis of oligomeric‑state changes, and intracellular target‑engagement studies.

Beyond glycolysis, HMLT intervention led to significant alterations in the lipid profile, characterized by notable changes in the levels of various phospholipids and lysophospholipids such as PC (18:2/0:0), PE (19:2/0:0), LysoPC (18:3/0:0), LysoPS (16:0/0:0), LysoPC (15:0). The observed increase in phospholipids suggests that HMLT triggers a reprogramming of lipid metabolism in macrophages. This indicates that lipid metabolism is in a highly dynamic state, characterized by continuous synthesis, hydrolysis, and remodeling. Consequently, this dynamic process provides the necessary lipid basis for the transition between inflammatory and anti-inflammatory states.

It should be noted that the animal experiments in this study employed a prophylactic administration regimen (HMLT administered 1 h before and 2 h after the first cerulein injection). This helps validate the intervention potential of the candidate molecule at the early onset of acute inflammation. Although a therapeutic administration regimen is more clinically relevant, the current prophylactic administration results already provide important proof-of-concept and mechanistic clues for the subsequent development of HMLT.

In summary, our work provides significant insights into the design of novel anti-inflammatory peptides by applying histidine substitution to a melittin-based scaffold. While we focused on elucidating its effects on macrophage polarization and metabolic reprogramming, we acknowledge the complex pathophysiology of SAP, wherein actions on acinar cells are also relevant. Our preliminary data showing reduced acinar cell cytotoxicity for HMLT compared to MLT further supports its improved safety profile.

Looking forward, several key research avenues emerge from this study. A critical next step will be to elucidate the precise molecular target(s) of HMLT. While prior studies suggest potential interactions with pattern-recognition receptors (e.g., TLR4/TLR2) or components of the NF-κB pathway, direct binding validation using techniques such as surface plasmon resonance (SPR) or cellular thermal shift assay (CETSA) is essential. Identifying the primary target will not only solidify the mechanistic foundation of HMLT but also directly inform the rational design of next-generation derivatives with improved specificity and potency. Beyond target identification, the generality of the histidine substitution strategy should be explored across a broader spectrum of peptide scaffolds to uncover more universal design principles. Furthermore, strategies to maintain or enhance membrane permeability, potentially through conjugation with cell-penetrating peptides warrant investigation to ensure optimal cellular uptake and efficacy. Concurrently, a deeper investigation into cellular and subcellular specificity is imperative to minimize off-target effects and enhance therapeutic precision. Ultimately, to substantiate the translational potential of HMLT and analogous engineered peptides, comprehensive preclinical and clinical studies will be necessary to fully evaluate their pharmacokinetics, safety, efficacy, and optimal dosing regimens.

## Conclusion

In conclusion, our study demonstrates that the histidine-substituted melittin analogue HMLT overcomes the cytotoxicity of natural peptides while maintaining potent anti-inflammatory effects. HMLT exerts its therapeutic action through dual mechanisms: mitigating oxidative stress by preserving mitochondrial function, reducing ROS, and enhancing endogenous antioxidants; and reprogramming macrophage metabolism by suppressing glycolysis. These coordinated actions significantly attenuate pancreatic damage and inflammation in SAP, highlighting HMLT’s translational potential as a novel therapeutic agent and validating the targeting of redox-metabolic crosstalk in macrophages as a promising strategy for SAP treatment.

## Supplementary Information

Below is the link to the electronic supplementary material.

Supplementary Material 1 (XLSX 10.2 KB)

Supplementary Material 2 (XLSX 1.69 MB)

Supplementary Material 3 (XLSX 1.71 MB)

Supplementary file 4 (PNG 87.1 KB)

Supplementary Material 4 (TIF 125 KB)

Supplementary Material 5 (DOCX 2.25 MB)

## Data Availability

Data is provided within the manuscript or supplementary information files.
