# Supplementary figures and images for: A Melittin-Derived Lead Compound Ameliorates Severe Acute Pancreatitis by Restoring Oxidative Homeostasis and Macrophage Metabolism
Source: Inflammation. 2026 Jan 22;49(1):59. doi: 10.1007/s10753-025-02444-9 (PMC12883531; doi:10.1007/s10753-025-02444-9)

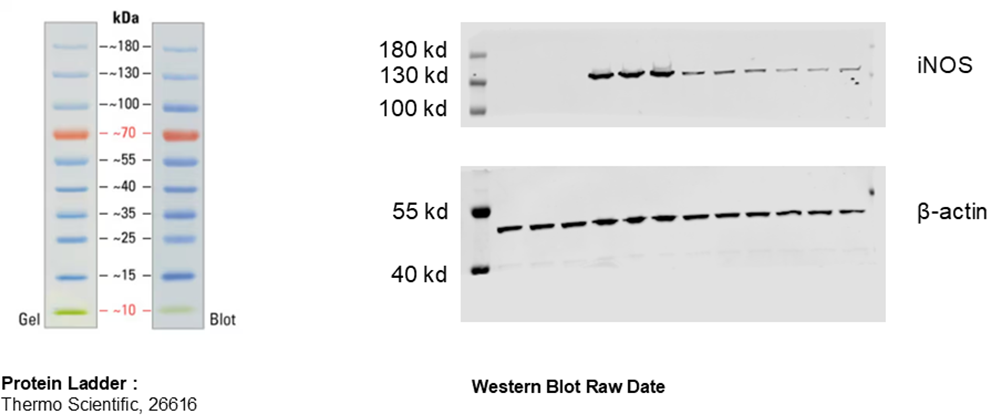

Supplement: Supplementary file 4 — (PNG 87.1 KB) [file 10753_2025_2444_Fig9_ESM.png]

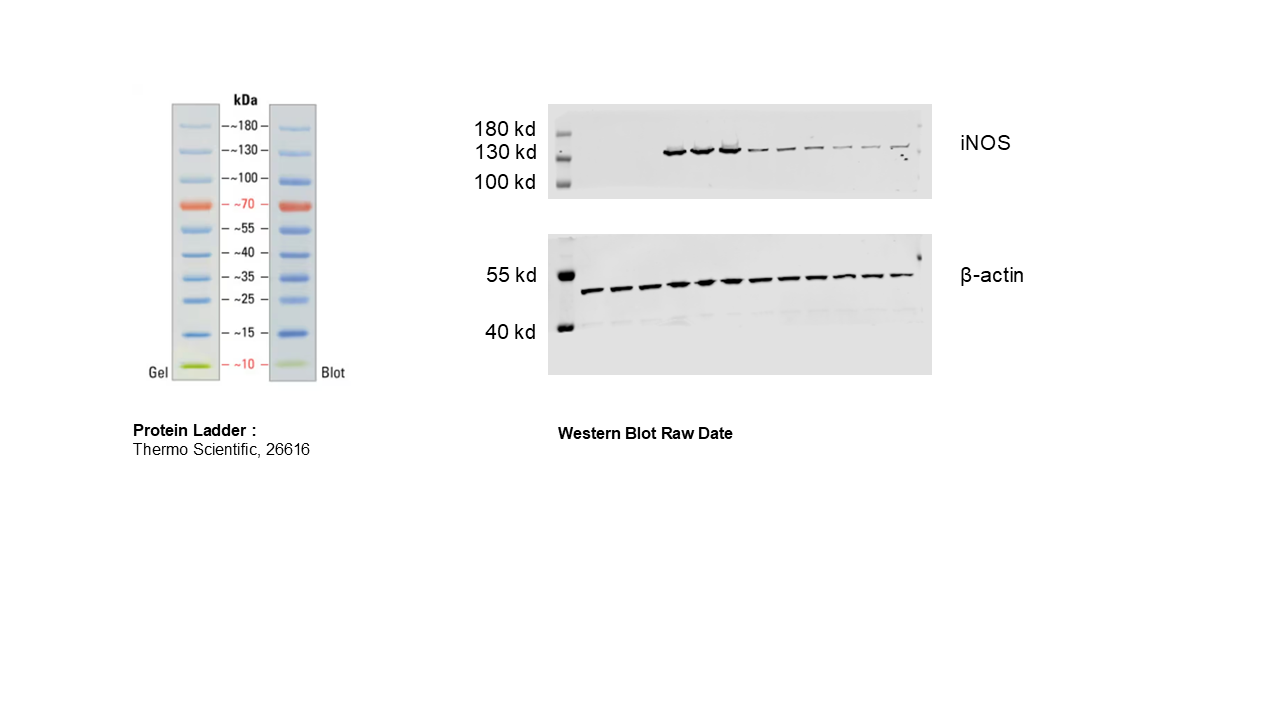

Supplement: Supplementary file 5 — Supplementary Material 4 (TIF 125 KB) [file 10753_2025_2444_MOESM4_ESM.tif]
