# Supplementary material for: A Melittin-Derived Lead Compound Ameliorates Severe Acute Pancreatitis by Restoring Oxidative Homeostasis and Macrophage Metabolism
Source: Inflammation. 2026 Jan 22;49(1):59. doi: 10.1007/s10753-025-02444-9 (PMC12883531; doi:10.1007/s10753-025-02444-9)
Supplement: Supplementary file 6 — Supplementary Material 5 (DOCX 2.25 MB) [file 10753_2025_2444_MOESM5_ESM.docx]

**Supporting Information**

**A Melittin-Derived Lead Compound Ameliorates Severe Acute Pancreatitis by Restoring Oxidative Homeostasis and Macrophage Metabolism**

**Table of Contents**

*Supplemental* *Methods*

DPPH radical scavenging activity…………………………………………………….…………S2

*Supplemental Figures*

Figure S1. RP-HPLC chromatogram and corresponding peak table of MLT…………………S2

Figure S2. Mass spectrometric identification of MLT..………………………………………S3

Figure S3. RP-HPLC chromatogram and corresponding peak table of MLT…………………S4

Figure S4. Mass spectrometric identification of MLT.………………………………………S5

Figure S5. Cytotoxicity of MLT and HMLT in murine pancreatic acinar 266-6 cells…………S6

Figure S6. WB images of iNOS..……………………………………………………….………S6

Figure S7. The ameliorative effect of HMLT on SAP.…………………………………………S7

Figure S8. PCA Score Plot………………………………………………………………………S7

Figure S9. The DPPH radical scavenging activity.……………………………………….………S8

**Supplemental Methods**

*DPPH radical scavenging activity*

Samples ranging from 0 to 256 μM were tested for their antioxidant capacity using the DPPH assay. Specifically, 0.1 mL of each sample was mixed with 0.1 mL of a 0.1 mM DPPH solution in alcohol, and the mixture was left to stand for 30 min at room temperature before measuring the absorbance at 517 nm (A_sample_). As a control, 0.1 mL of distilled water was mixed with 0.1 mL of the 0.1 mM DPPH solution in alcohol and subjected to the same procedure, with absorbance measured after 30 min (A_control_). Additionally, 0.1 mL of each sample was mixed with 0.1 mL of anhydrous ethanol, and the mixture was also allowed to stand for 30 minutes at room temperature before the absorbance at 517 nm (A_blank_)was recorded.

DPPH scavenging efficiency = (1- (A_sample_ - A_blank_) / A_control_) × 100%

**Supplemental Figures**


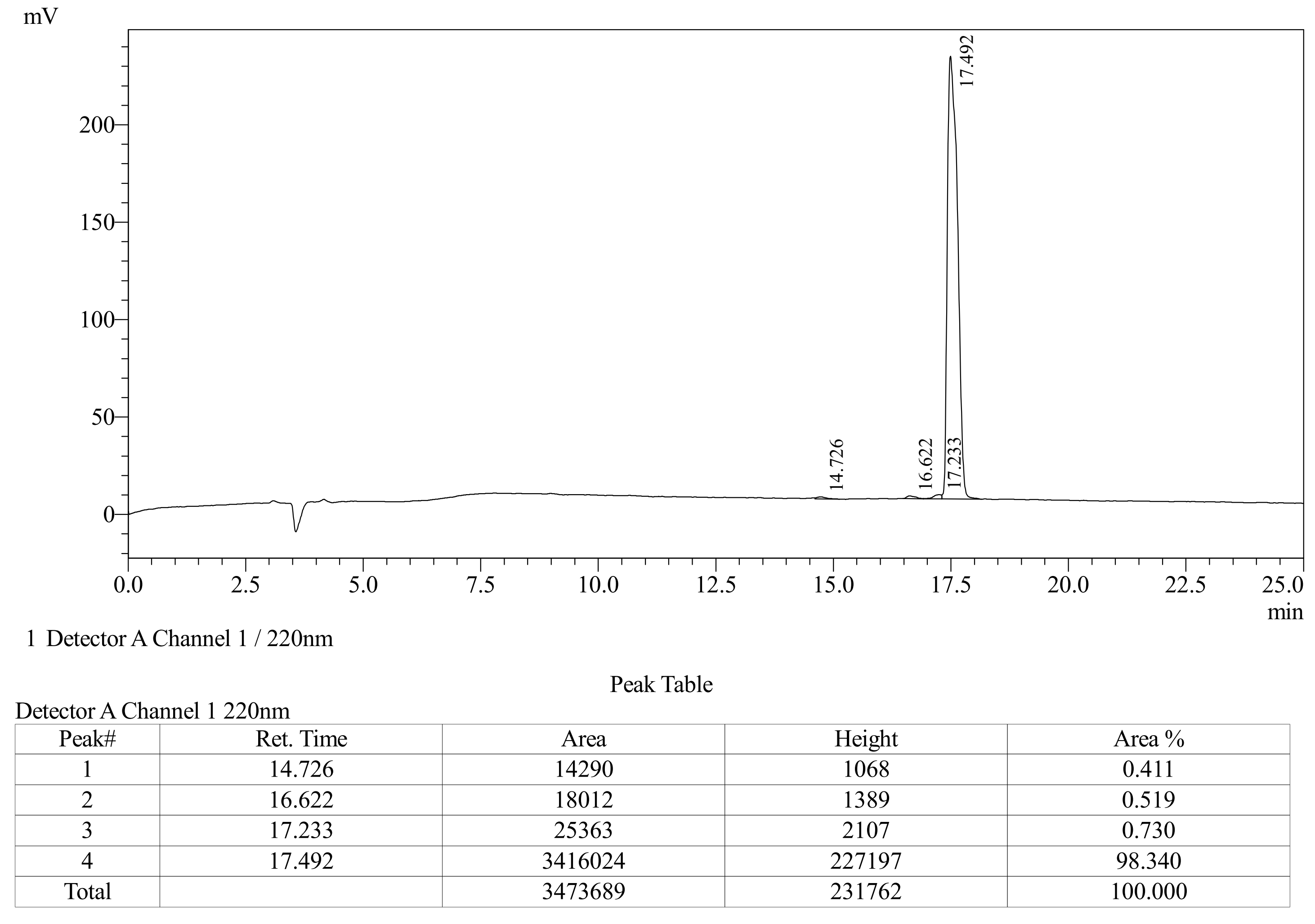


**Fig. S1 RP-HPLC chromatogram and corresponding peak table of MLT.** The detection wavelength was set at 220 nm, using an Inertsil ODS-3 column (4.6 × 250 mm). Mobile phase A consisted of 0.065 % trifluoroacetic acid in 100 % water (v/v), and mobile phase B consisted of 0.05 % trifluoroacetic acid in 100 % acetonitrile (v/v). The total flow rate was 1 mL/min.


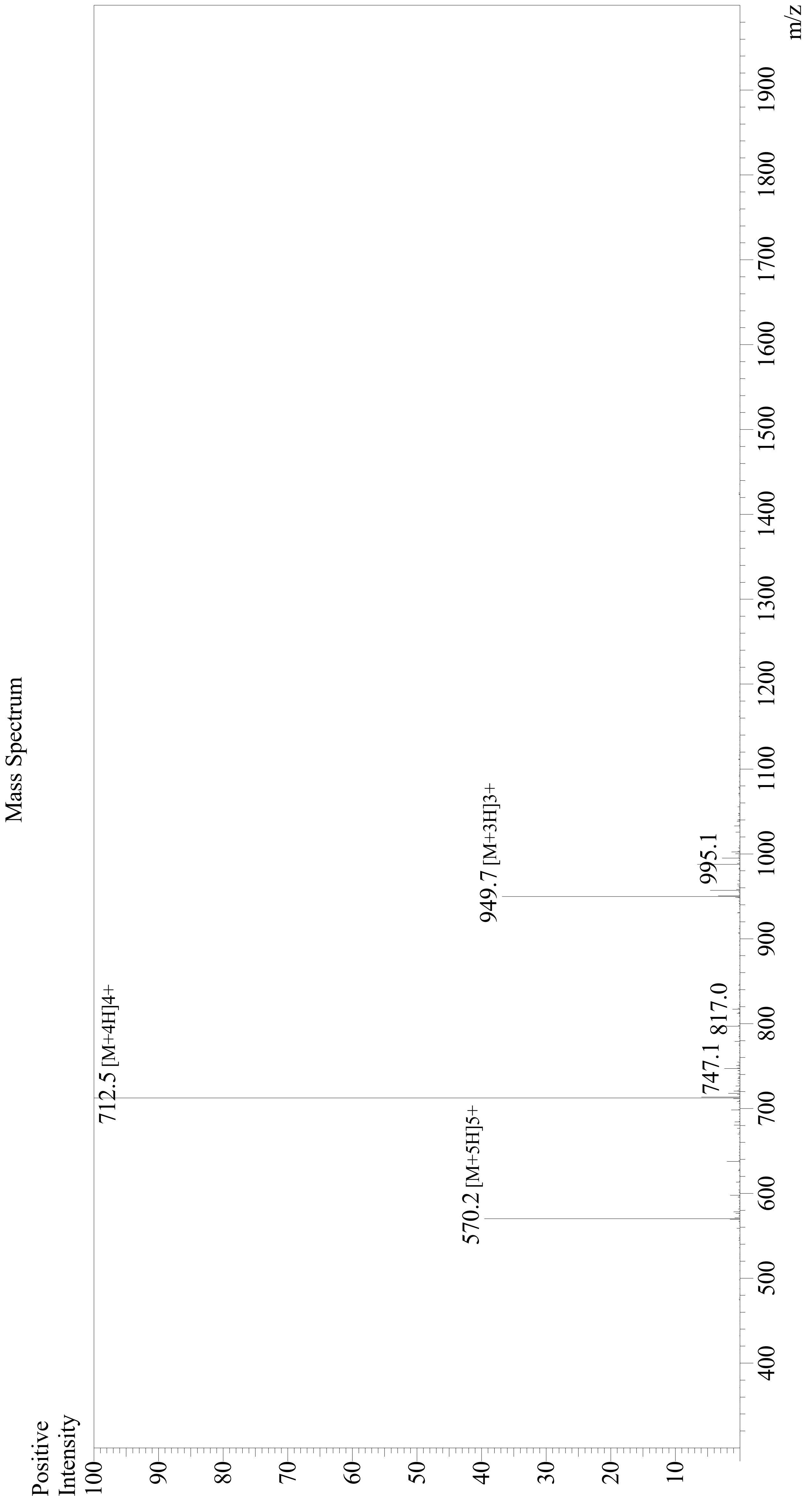


**Fig. S2 Mass spectrometric identification of MLT.** The theoretical molecular weight of MLT is 2846.48, while the detected molecular weight was 2846.0. The analysis was performed using electrospray mass spectrometry (ESI-MS).


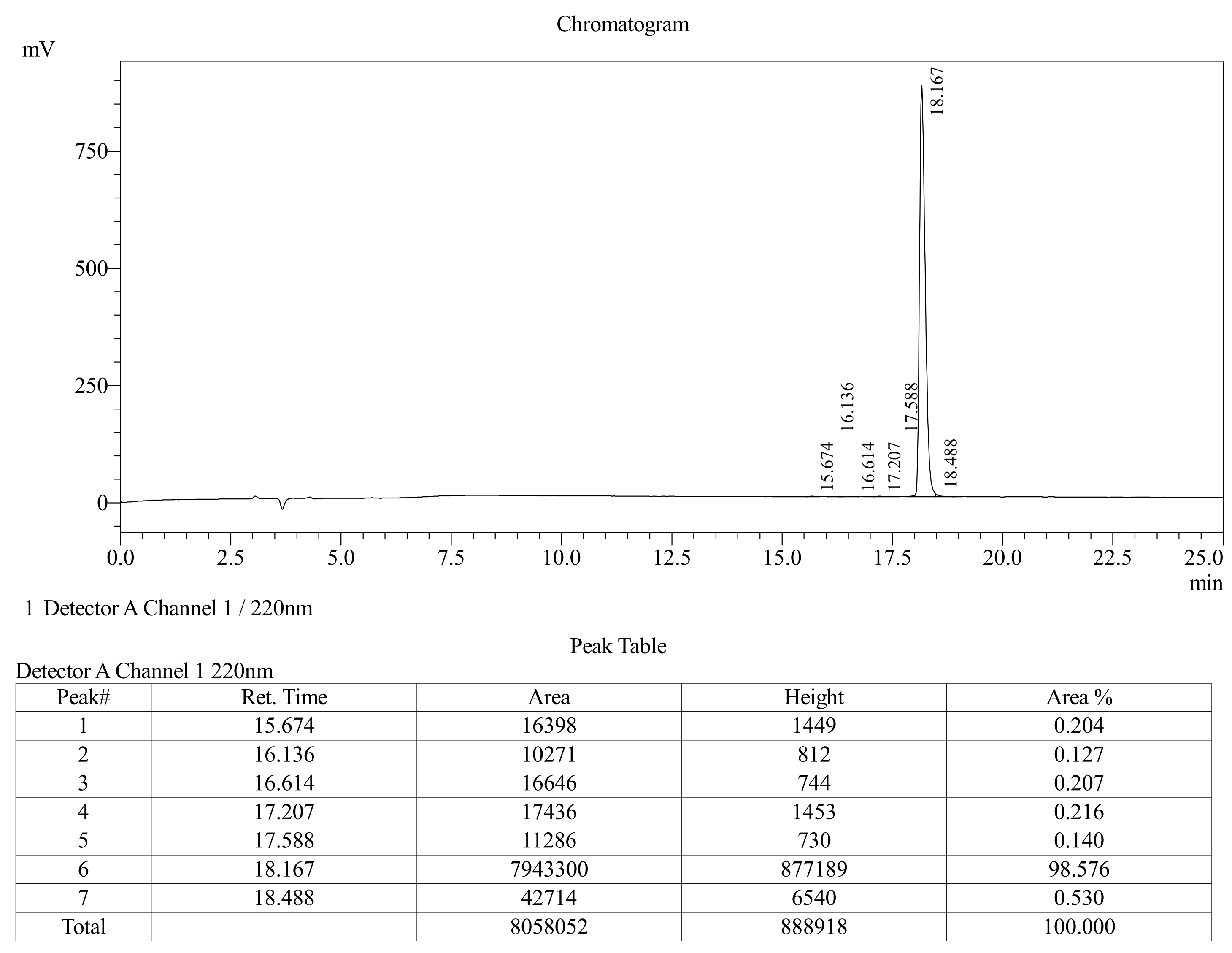


**Fig. S3 RP-HPLC chromatogram and corresponding peak table of HMLT.** The detection wavelength was set at 220 nm, using an Inertsil ODS-3 column (4.6 × 250 mm). Mobile phase A consisted of 0.065 % trifluoroacetic acid in 100 % water (v/v), and mobile phase B consisted of 0.05 % trifluoroacetic acid in 100 % acetonitrile (v/v). The total flow rate was 1 mL/min


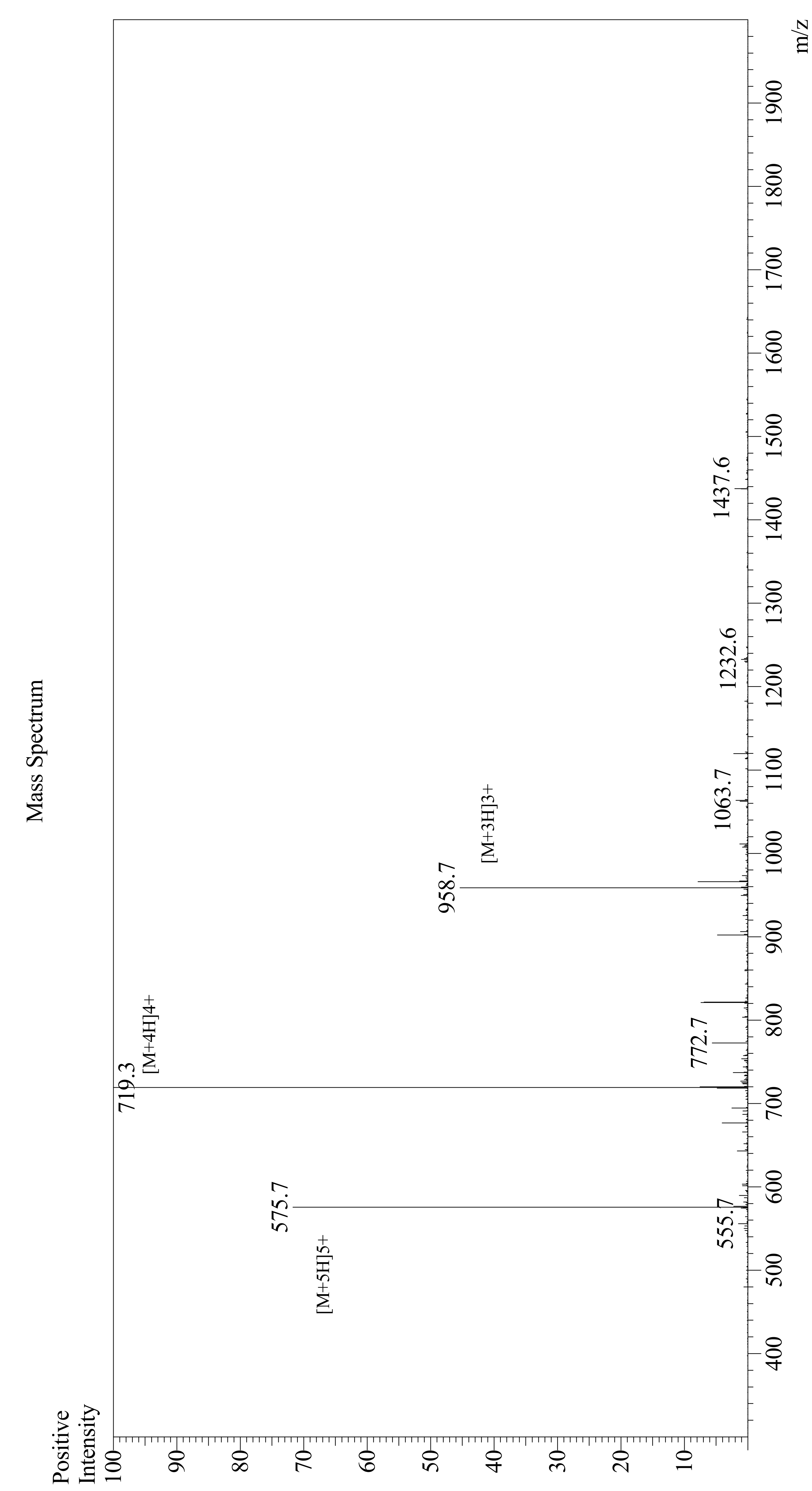


**Fig. S4 Mass spectrometric identification of HMLT.** The theoretical molecular weight of MLT is 2873.38, while the detected molecular weight was 2873.2. The analysis was performed using ESI-MS.

**
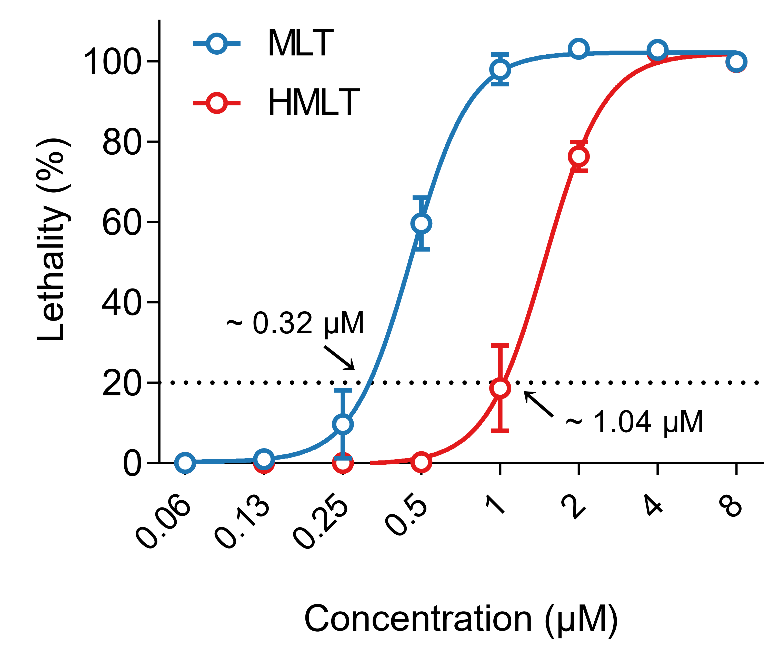
Fig. S5 Cytotoxicity of MLT and HMLT in murine pancreatic acinar 266-6 cells.** Cells were treated with various concentrations of peptides for 24 h at 37 °C, followed by incubation with CCK-8 reagent for an additional 3 h.


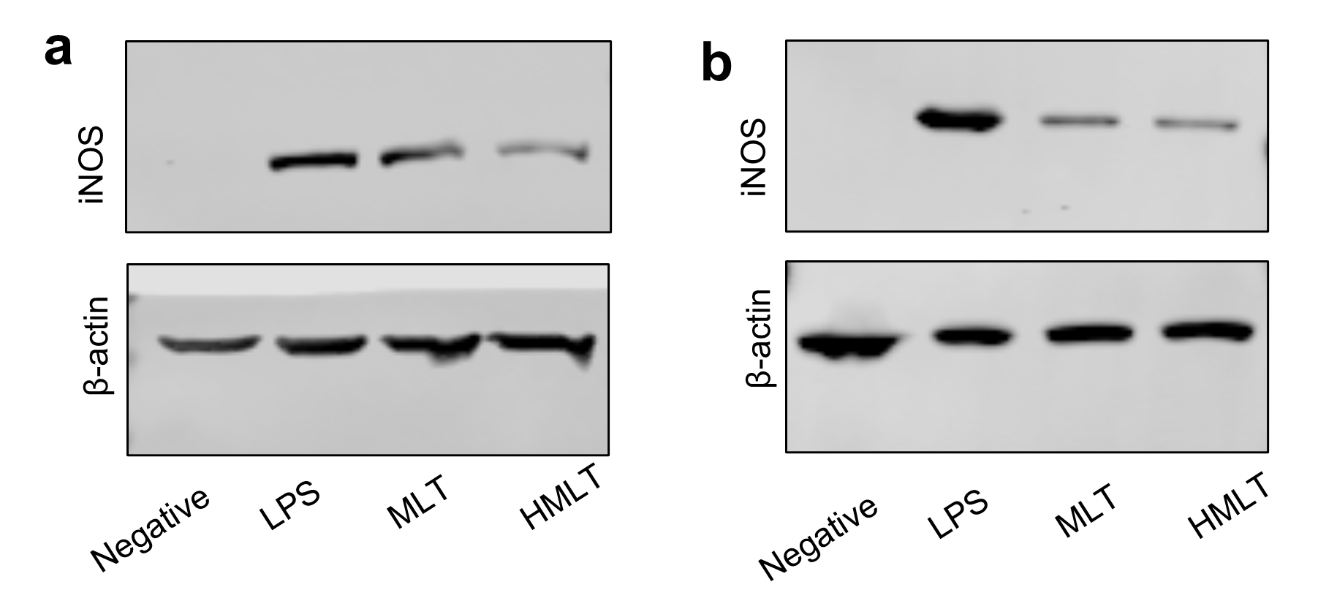


**Fig. S6 WB images of iNOS. a** and **b** represent two independent replicate experiments.


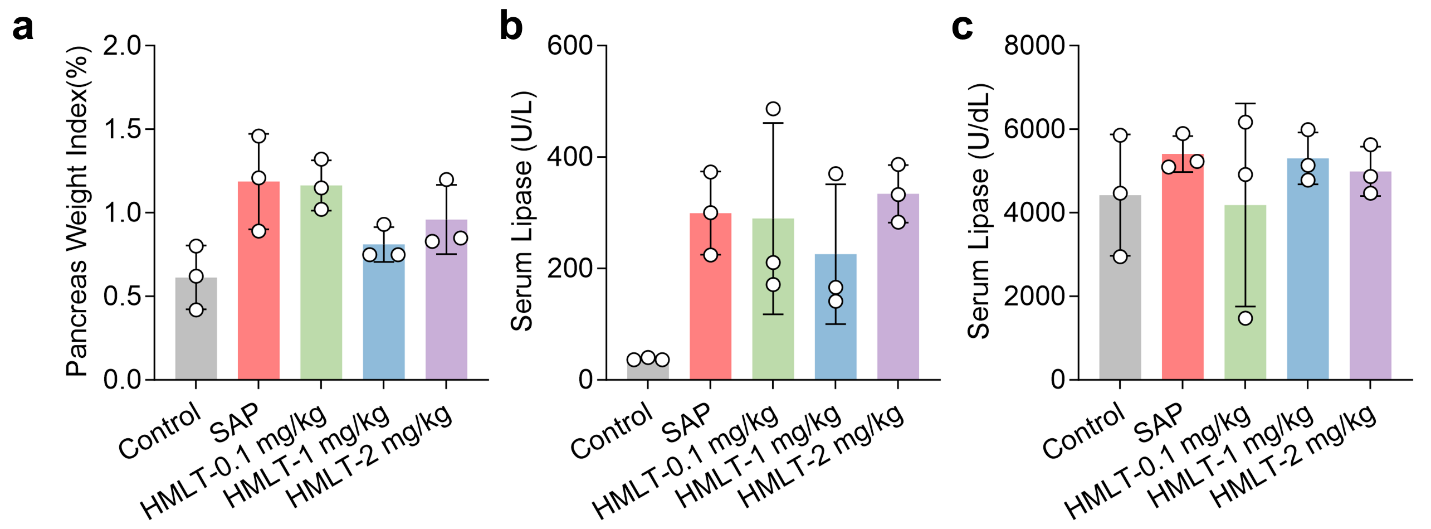


**Fig. S7 The ameliorative effect of HMLT on SAP. a** Bar plots present the quantification of pancreas weight index in in different groups (n = 3). **b, c** Bar plots present the quantification of serum lipase and amylase in different groups (n = 3).


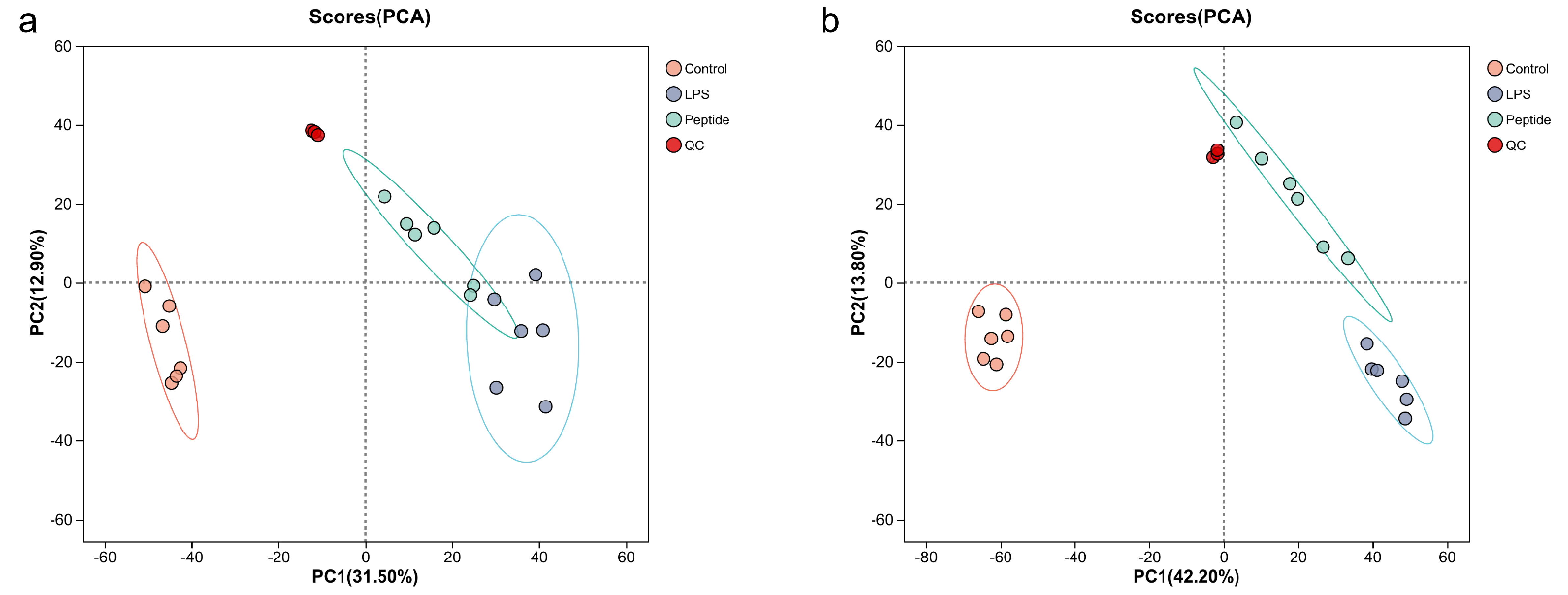


**Fig. S8** **PCA Score Plot.** Following dimensionality reduction, samples are plotted as relative coordinates on principal components P1 and P2. The proximity of these points reflects sample similarity, with closer points indicating higher similarity and more distant points signifying greater differences. **a** PCA score plots of overall samples in positive mode; **b** PCA score plots of overall samples in negative mode.

**Fig. S9** **The DPPH radical scavenging activity.** Samples (0-256 μM) were assessed for antioxidant activity via DPPH assay, with absorbance measured at 517 nm after 30 min incubation. Controls and ethanol mixtures were also tested.
